# Deep spatial profiling of Venezuelan equine encephalitis virus reveals increased genetic diversity amidst neuroinflammation and cell death during brain infection

**DOI:** 10.1128/jvi.00827-23

**Published:** 2023-08-10

**Authors:** Evan P. Williams, Yi Xue, Jasper Lee, Elizabeth A. Fitzpatrick, Ying Kong, Walter Reichard, Haley Writt, Colleen B. Jonsson

**Affiliations:** 1 Department of Microbiology, Immunology and Biochemistry, University of Tennessee Health Science Center, Memphis, Tennessee, USA; 2 Regional Biocontainment Laboratory, University of Tennessee Health Science Center, Memphis, Tennessee, USA; 3 Institute for the Study of Host-Pathogen Systems, University of Tennessee Health Science Center, Memphis, Tennessee, USA; Cornell University Baker Institute for Animal Health, Ithaca, New York, USA

**Keywords:** immune response, brain, pathology, nonsynonymous mutations, RNA sequencing, mice, Venezuelan encephalitis virus

## Abstract

Venezuelan equine encephalitis virus (VEEV) causes a febrile illness that can progress to neurological disease with the possibility of death in human cases. The evaluation and optimization of therapeutics that target brain infections demands knowledge of the host’s response to VEEV, the dynamics of infection, and the potential for within-host evolution of the virus. We hypothesized that selective pressures during infection of the brain may differ temporally and spatially and so we investigated the dynamics of the host response, viral transcript levels, and genetic variation of VEEV TC-83 in eight areas of the brain in mice over 7 days post-infection (dpi). Viral replication increased throughout the brain until 5–6 dpi and decreased thereafter with neurons as the main site of viral replication. Low levels of genetic diversity were noted on 1 dpi and were followed by an expansion in the genetic diversity of VEEV and nonsynonymous (Ns) mutations that peaked by 5 dpi. The pro-inflammatory response and the influx of immune cells mirrored the levels of virus and correlated with substantial damage to neurons by 5 dpi and increased activation of microglial cells and astrocytes. The prevalence and dynamics of Ns mutations suggest that the VEEV is under selection within the brain and that progressive neuroinflammation may play a role in acting as a selective pressure.

**IMPORTANCE:**

Treatment of encephalitis in humans caused by Venezuelan equine encephalitis virus (VEEV) from natural or aerosol exposure is not available, and hence, there is a great interest to address this gap. In contrast to natural infections, therapeutic treatment of infections from aerosol exposure will require fast-acting drugs that rapidly penetrate the blood-brain barrier, engage sites of infection in the brain and mitigate the emergence of drug resistance. Therefore, it is important to understand not only VEEV pathogenesis, but the trafficking of the viral population within the brain, the potential for within-host evolution of the virus, and how VEEV might evolve resistance.

## INTRODUCTION

Venezuelan equine encephalitis virus (VEEV) is a mosquito-borne, New World *Alphavirus* that has caused a number of epidemic/epizootic outbreaks of disease in humans and equines in the past century in the Americas (1, 2). Epizootic outbreaks of VEEV in human populations occur when mosquitos transmit the virus from small mammals to equines, which function as an intermediate, amplifying host for the virus. In humans, VEE is characterized by a febrile illness, symptoms of which include fever, malaise, myalgia, and headache (3 - 5). In some cases, particularly in children and older adults, VEE can progress to neurological disease, with symptoms including convulsions, seizures, stupor, coma, and the possibility of death (4, 6). Pathogenesis comes from lymphocyte destruction, endothelial cell injury, and encephalitis (5, 7 - 9). The development of VEEV as a bioweapon during the Cold War resulted in its designation as a select agent by the Centers for Disease Control and Prevention and as a Category B priority pathogen by the National Institutes of Health (10, 11). The biothreat of VEEV rests in its ability to grow to high titers and its ability to be disseminated by aerosol exposure. Moreover, in aerosol or intranasal infections, VEEV reaches the olfactory nerves, and then the brain, much more rapidly compared to a natural infection via a mosquito bite (12). Treatment of infection and disease in humans caused by VEEV from a natural or an aerosol exposure is not available, and hence, there is great interest to address this gap. Treatments for aerosol infections will require drugs that rapidly penetrate the blood-brain barrier, engage all areas of the brain to mitigate and prevent viral replication and dissemination, eliminating any potential for the emergence of drug resistance of the virus. Therefore, it is important to understand not only the pathogenesis, but to define the areas of the brain to which the virus traffics, the associated virus population structure, and underlying spatial dynamics. If the genetic variation of a virus changes over time, this may affect the trajectory of resistance.

Our understanding of the dynamics of encephalitis caused by VEEV, progression of infection, disease, and host response, comes predominantly from studies in mice [e.g., see references (10, 13 - 21)], since infection of cynomolgus monkeys is not lethal, although key neurological and other disease symptoms are observed (13, 22). Neurological disease occurs when VEEV penetrates the central nervous system (CNS) by accessing olfactory neurons in the olfactory tract, which are easily accessible from either blood vessels or from nerve endings of olfactory receptor neurons that cross the nasal olfactory epithelium (21). From here, VEEV quickly reaches the neurons within the olfactory bulb and spreads through the rest of the brain. Tissue damage within the CNS of mice occurs in the form of apoptosis and necrosis of infected neurons, perivascular cuffing, and meningitis in areas of the brain that the virus has reached (9). The immune response is important for controlling VEEV infection but may also contribute to encephalitis during neurologic disease (23). In most mouse models of VEEV, there is extensive infiltration of T cells, B cells, NK cells, neutrophils, and monocytes into the CNS which is accompanied by the increased expression of pro-inflammatory cytokines and chemokines. Several lines of evidence have suggested that CD4^+^ T cells are protective (10, 24), whereas NK cells have been reported to increase pathology (16).

To date, we have no knowledge of the within-host population structure and genetic variation of the VEEV genome during infection and disease progression within the brain. Like other viruses with an RNA-based genome, VEEV has high mutation rates during replication as its RNA-dependent RNA polymerase has a high error rate (25, 26). The quasispecies hypothesis suggests that error-prone replication generates a population of many individually distinct viruses that contribute to an overall phenotype (27). The resulting genetic diversity of the population of VEEV may then ensure successful infection of multiple hosts, i.e., mammals and mosquitos as well as the ability to successfully infect multiple organs in mammals (28). For example, in mosquitos, its high mutation rate provides a mechanism to regain fitness after the virus undergoes a bottleneck at the midgut resulting in a small number of infected cells (29).

We hypothesized that the host response during infection of the brain may differ temporally and spatially and so we investigated the host response, population dynamics, and genetic variation of VEEV TC-83 in eight areas of the brain over 7 days (days 1–7 post-infection, dpi) following its intranasal exposure of C3H/HeN mice. We focused on the main olfactory bulb, piriform cortex, striatum, motor cortex, sensory cortex, hippocampus, thalamus, and cerebellum. VEEV TC-83 infection is lethal in the mouse strain, C3H/HeN, when administered intranasally (i.n.), intracranially, or by aerosol exposure, and mice become moribund from 7 to 9 dpi (30 - 33). We noted a low level of genetic diversity of VEEV TC-83 on 1 dpi coinciding with infection of the main olfactory bulb. The amount of genetic diversity as revealed by single-nucleotide polymorphisms (SNPs) of the viral genome and viral transcript levels increased over time, but there was no correlation between them. The number of nonsynonymous (Ns) mutations peaked at 5 dpi in numerous areas of the brain and mirrored the dynamics observed in the distribution of the viral glycoprotein (GP) by immunohistochemistry and neuroinflammatory responses by RNA sequencing (RNA-Seq). Immune cell composition was characterized by an influx of monocytes and macrophages and activation of microglial cells. The prevalence and dynamics of Ns mutations suggest that the VEEV genome is under selection and correlate with the host’s pro-inflammatory response and cell death.

## MATERIALS AND METHODS

### Cells and viral culture

Vero 76 cells (ATCC CRL-1587) were maintained at 37°C and 5% CO_2_ in complete medium containing Dulbecco’s modified Eagle medium with Hi-glucose and L-glutamine supplemented with 10% fetal bovine serum (FBS) and 1% penicillin-streptomycin. VEEV TC-83 was obtained from Connie Schmaljohn (United States Army Medical Research Institute of Infectious Diseases, Maryland). Virus seed stocks were amplified in Bovine hamster kidney (BHK)cells in minimum essential medium with Earle’s salts Eagle's minimum essential medium (EMEM) with 2% FBS and 1% penicillin-streptomycin. All cell culture reagents were purchased from Thermo Fisher Scientific unless otherwise specified. VEEV TC-83 was sequenced as a reference genome as previously reported (34).

### Plaque assay

Twelve-well plates were seeded with 2.5 × 10^5^ Vero 76 cells and a series of 10-fold dilutions of virus samples were made to measure titer as described in reference (35).

### General animal study information and compliance for all studies

C3H/HeN mice were purchased from Charles River Laboratories for all studies. In all studies, mice were inoculated i.n. with 1 × 10^7^ plaque-forming units (PFU) of VEEV TC-83 or mock inoculated with Dulbecco’s phosphate-buffered saline (PBS) using 15 µL per naris. After inoculation, all mice were weighed daily and checked twice daily for clinical signs and morbidity. All mice were humanely sacrificed using isoflurane, followed by cervical dislocation. All animal studies and methods were conducted under the protocol 18-044 or 21-0258, authorized by the Institutional Animal Care and Use Committee at the University of Tennessee Health Science Center.

### Mouse study design and sample collection for RNA-Seq

Twenty-eight, 5- to 6-week-old, female, C3H/HeN mice were randomly assigned to groups. Each group of VEEV TC-83 infected mice (four mice per group) were humanely sacrificed at 1, 3, 5, 6, or 7 dpi. Mock-inoculated mice were sacrificed on 1 and 5 dpi. Following euthanasia, the whole brain, including the olfactory bulb, was removed from each mouse. Each brain was separated into eight portions: main olfactory bulb, piriform cortex, caudate-putamen, motor cortex, sensory cortex, thalamus, hippocampus, and cerebellum. Each section was homogenized in 1 mL of lysis buffer from the MagMAX mirVana Total RNA Isolation Kit (Thermo Fisher Scientific). Total RNA was isolated from each brain section using the MagMAX mirVana Total RNA Isolation Kit and the KingFisher Flex System (Thermo Fisher Scientific).

### RNA-Seq

Sequencing of total RNA was performed by Azenta Life Sciences. Total RNA was quantified and qualified using a Qubit (Thermo Fisher Scientific) and TapeStation (Agilent), whereafter sequencing libraries were constructed by mRNA enrichment using NEBNext Ultra II RNA Kits (New England Biolabs). Constructed libraries were sequenced on Illumina’s HiSeq platform (2 × 150, 40 million pair-end reads per sample). Sequence read results were processed with CLC Genomics Workbench (Qiagen, v23). Sequencing reads from each sample underwent trimming and filtering using default parameters. Read mapping was conducted using default parameters and performed to a concatenated genome genome consisting of *Mus musculus* (Ensembl, GRCm38.108) and sequenced lab strain of VEEV TC-83. Gene count results were used to assess differential gene expression using DESeq2 and mock-inoculated mice defined baseline gene expression. Determined differentially expressed genes (log_2_ fold change ≤−2 and ≥2; false discovery rate ≤0.05) were used to assess canonical pathway activation as well as perform comparison analysis using Ingenuity Pathway Analysis (Qiagen, April 2022 release). RNA-Seq data were uploaded to the NIH Gene Expression Omnibus (GSE213725).

### Assessment of virus mutations

Sequencing reads (described above) that mapped to the genome sequence of our lab seed stock VEEV TC-83 was used to assess virus mutations using CLC Genomics Workbench (Qiagen, v23). Viral SNPs were identified using low-frequency variant detection was used with the following parameters, minimum coverage = 100, minimum count = 50, and minimum frequency = 1.0%. Ns mutations were determined by using the Amino Acid Change tool to filter away SNPs that did not result in nonsynonymous changes.

### Mouse study design and sample collection for immunohistochemistry

Twenty-eight, 5- to 6-week-old, female, C3H/HeN mice were randomly assigned to groups based on day of sacrifice, i.e., 1, 2, 3, 4, 5, 6, and 7 dpi, and VEEV TC-83 or mock inoculated. Following euthanasia, the whole brain with the olfactory bulb was removed from each mouse and fixed in 20 mL of 10% formalin for 24 h at 4°C. One sagittal section of each brain was made and then the tissue was cryoprotected with 30% sucrose in 0.1 M phosphate buffer pH 7.4. Sagittal sections of brain were made with a Leica CM3050 at 40 µM thickness. To collect nasal turbinate, each mouse skull was fixed with 10% buffered formalin, dissected, decalcified with 10% ethylenediaminetetraacetic acid (EDTA), and embedded in paraffin for samples taken on 1, 3, and 5 dpi. About 4 μm sections were cut using a Leica microtome.

### Immunohistochemical staining of mouse brains

For immunohistochemical (IHC) staining and imaging of cryosections, brain sections were washed with PBS and endogenous peroxidases were quenched with 1% hydrogen peroxide in PBS for 5 min, rinsed with PBS three times and blocked with 1% bovine serum albumin in PBS and 0.3% Triton X-100. Floating sections were incubated with goat anti-VEE GP antibody (gift from Kurt Kamrud, AlphaVax, NC) at a 1:12,000 dilution for 2 days at 4°C on a shaker, for rabbit AIF-1Iba-1 antibody (NBP2-19019, Novus Biologicals), with dilution of 1:1,000, rabbit anti-GFAP antibody (NB300-141, Novus Biologicals) with dilution of 1:2,000, rinsed with PBS three times and incubated with biotinylated rabbit anti-goat IgG at 1:200 (BA-5000, Vector Laboratories) or biotinylated horse anti-rabbit IgG (BA-1100, Vector Laboratories) for overnight. After rinsing three times with PBS, sections were incubated with avidin-biotin complex (ABC) using the Vectastain ABC Elite Kit PK-6100 (Vector Laboratories, USA) at a 1:100 dilution and developed with 3,3ʹ-diaminobenzidine (DAB) (Sigma-Aldrich, USA). Sections were mounted onto gelatin-coated glass slides and air dried, counterstained with methyl green solution or without counterstain, dehydrated through serial alcohol rinses and xylene and then mounted using Permount Mounting Media (Thermo Fisher Scientific).

For IHC with paraffin-embedded sections, we used heat-induced antigen retrieval for OMP with Tris/EDTA buffer pH 9.0 (H3301) and for LDLRAD3 we used citric buffer pH 6 (H3300). Both products are from Vector Laboratories. For OMP (rabbit anti-Olfactory Marker Protein/OMP antibody, EPR19190, ab183947, Abcam), we used a 1:2,000 dilution for LDLRAD3 (rabbit anti-LDLRAD3 antibody, NBP1-86261, Novus Biologicals), we used a 1:50 dilution, and for the VEEV GP, we used a 1:1,000 dilution. Incubations with primary antibodies were done overnight followed by ABC method as described above.

### Histopathological and immunohistochemical staining of nasal cavity

About 5 µm sections of formalin-fixed nasal turbinates were cut, mounted on Fisherbrand Superfrost Plus Microscope Slides (Fisher Scientific), and stained with hematoxylin and eosin (H&E). For paraffin-embedded nasal mucosa/epithelium sections, heat-induced antigen retrieval was performed. Sections were incubated with goat anti-VEEV GP antibody at a 1:1,000 dilution and treated using ABC and DAB as described above.

### Double immunofluorescent labeling of viral antigen and Iba-1 and GFAP in brain sections

Cryostat sections of mock and VEEV infected mice brains were incubated with a cocktail of goat anti-VEEV capsid antibody (gift from Kurt Kamrud, AlphaVax, NC) at 1:8,000 dilution with rabbit anti-Iba-1 antibody at 1:1,000 (NBP2-19019, Novus Biologicals) or rabbit anti-GFAP at 1:2,000 NB300-141, Novus Biologicals) for 2 days, after which slide sections were rinsed with PBS, and then incubated with a cocktail of Donkey anti-Rabbit IgG (H + L) Alexa Fluor 488 (Invitrogen) or Donkey anti-Goat IgG (H + L) Secondary Antibody, Alexa Fluor 594 (Invitrogen) at 1:500 overnight. Slides were rinsed with PBS counterstained with DAPI, mounted and coverslip with Fluoromount-G (SouthernBiotech, USA). Images captured with Zeiss 710 confocal microscope.

### Digital imaging and processing

All slides were scanned with an Olympus VS200 by scanning slides with a 40× objective. Images were captured and processed with the OlyVia v. 3.2 software.

### Mouse study design and sample collection for flow cytometry

Twenty-four, 5- to 6-week-old, female, C3H/HeN mice (*n* = 4/day) were humanely sacrificed at 1, 4, or 7 dpi. Whole brains, including olfactory bulbs, were harvested and brain cells were isolated using 0.1 mg/mL Collagenase D and 5 µg/mL DNase for 30 min at 37°C. The cells were filtered through a 70-µm cell strainer and then resuspended in 37% Percoll, followed by centrifugation at 860 × *g*.

### Flow cytometry

The cell pellet obtained from Percoll as stated above was resuspended in fluorescence-activated cell sorting (FACS) buffer and cell surface flow cytometry performed using fluorochrome-conjugated antibodies to CD11b (clone M1/700), CD206 (clone C068C2), CD45 (clone 30-F11), CD4 (clone RM4-5), TcRβ(clone H57-597), CD80 (clone 16-10A1), or CD69 (clone H1.2F3). All antibodies were from BioLegend, San Diego, CA, USA or BD Biosciences, Franklin Lake, NJ, USA. A minimum of 10,000 events/sample were collected on a Bio-Rad ZE5 Cell Analyzer (Bio-Rad, CA). The expression of cell surface markers was analyzed using FlowJo v10.7.2 software.

## RESULTS

### RNA sequencing of VEEV TC-83 in the brain reveals an early bottleneck followed by an expansion in nonsynonymous mutations

**Fig 1:**
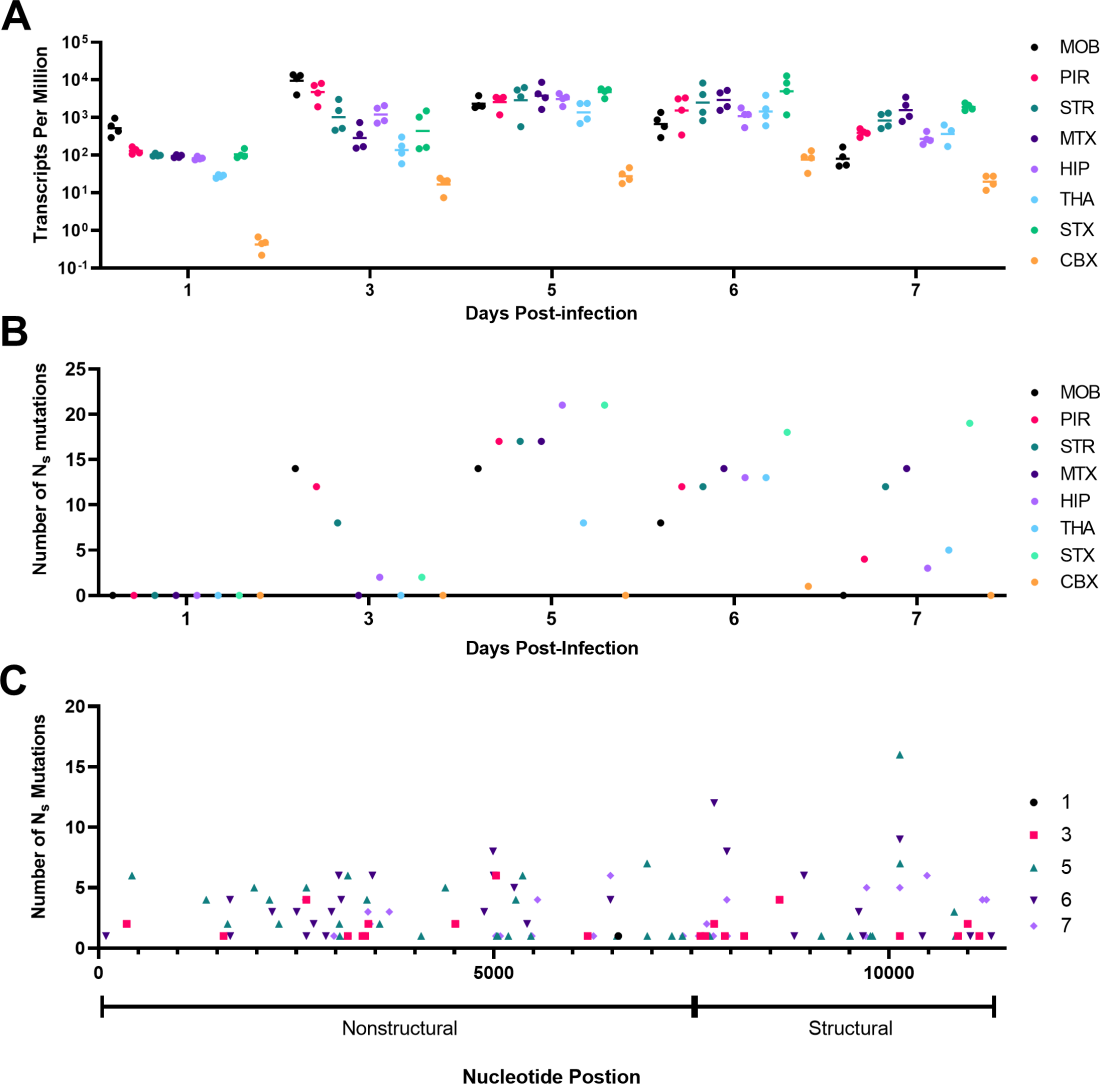
Longitudinal study of viral transcript abundance and nonsynonymous (Ns) mutations in brain following intranasal infection with VEEV TC-83. Twenty, 5- to 6-week-old mice were humanely sacrificed at 1, 3, 5, 6, or 7 dpi (*n* = 4/day) for brain sections were dissected for RNA-Seq as described in the Materials and Methods. The number of VEEV TC-83 transcripts per million or TPM (**A**) and the number of Ns mutations (**B**) are presented for each day for each of the eight areas of the brain. To identify Ns, variant calling was performed such that each variant had a minimum coverage of 100, a count of 50, and a frequency of 1%. (**C**) The number of Ns mutations discovered from all samples are presented in the context of the structural and nonstructural genes of the genome. CBX, cerebellum; dpi, days post-infection; HIP, hippocampus; MOB, main olfactory bulb; MTX, motor cortex; PIR, piriform cortex; STR, striatum; THA, thalamus.

To evaluate genomic transcript levels and potential SNPs of VEEV TC-83 during lethal infection of the brain, C3H/HeN mice were intranasally inoculated with 10^7^ PFU of VEEV TC-83 on day 0, and then mice were sacrificed on 1, 3, 5, 6, and 7 dpi. Each day, eight areas of the brain were dissected for RNA-Seq: the main olfactory bulb, piriform cortex, striatum, motor cortex, sensory cortex, thalamus, hippocampus, and cerebellum. The number of viral genome transcripts was normalized during library preparation and then by assessment of transcripts per million (TPM). The TPM values increased by 3 dpi for all areas of the brain, and by 5 dpi, the main olfactory bulb and piriform cortex showed a slight decrease that continued to 7 dpi (Fig. 1A). The striatum, motor cortex, sensory cortex, thalamus, hippocampus, and cerebellum all showed increases in TPM from 3 to 5 dpi. By 6 dpi, all areas of the brain except for cerebellum had similar levels of viral TPM and this was followed by a decrease by 7 dpi. The viral TPM in the cerebellum was significantly lower than all the other areas of the brain throughout all days.

The SNPs in each area of the brain were defined by comparison to the reference sequence from our laboratory seed stock of VEEV TC-83. We defined location, amino acid change, location, number of times observed, section of brain, and frequency of all Ns mutations (Table S1). A total number of 329 Ns mutations were discovered and plotted for each area on each day post-infection (Fig. 1B). Of the 329 Ns mutations, 102 were unique across all time points. Remarkably, no Ns mutations were detected at 1 dpi in any tissue, although viral TPM was extremely low in the cerebellum. By 3 dpi, notable increases in Ns were noted for olfactory bulb, striatum, and piriform cortex. By 5 dpi, most areas had increased Ns, but the cerebellum did not and mostly remained below detection through 7 dpi. The number of Ns in the sensory cortex remained high through 7 dpi. Except for thalamus, 5 dpi represented the peak of genetic diversity with 123 Ns mutations, of which 36 were unique (Table S1). No correlation was noted between the level of virus (TPM) and the number of Ns mutations in any section of the brain (Fig. S1). Prominent levels of subgenomic RNA were present in RNA-Seq analyses in all samples suggesting active replication in all areas of the brain (Fig. S2).

The distribution of Ns mutations over time was similar in the nonstructural and structural genes (Fig. 1C). Ns mutations were observed in every gene except for the 6K and E3. The viral gene that had the greatest number of Ns mutations was the nonstructural protein 2 (NSP2), with 100 of which 31 were unique (Table S1). This was followed by NSP3 (62, 19 unique), E1 (63, 13 unique), capsid (38, 12 unique), NSP4 (25, 8 unique), E2 (25, 11 unique), and finally NSP1 (16, 6 unique). Assessment by analysis of variance (ANOVA) of the location where the Ns mutations were observed across the genome of VEEV TC-83 showed that there was no difference between the number of Ns mutations found in the structural or nonstructural genes and this was true for all days studied (3 dpi adjusted *P* value = 0.7031; 5 dpi adjusted *P* value = 0.7031; 6 dpi adjusted *P* value = 0.7031; and 7 dpi adjusted *P* value = 0.7031).

Of the 102 unique Ns mutations (Table S1), only 6 (NSP2:D326E, NSP2:D502V, NSP4:K258T, capsid:G76R, capsid:I130F, and E1:Y46C) were observed across multiple days, brain portions, and mice. Two Ns mutations (NSP2:F286L and NSP3:T333I) were observed across multiple brain areas and multiple mice, and 49 Ns mutations were only observed in multiple brain areas of the same individual mouse but not on multiple days. Finally, we measured 47 Ns mutations in only one area of the brain.

### Spatial and temporal tracking of the VEEV TC-83 glycoprotein antigen in C3H/HeN from the nasal cavity to the brain

**Fig 2:**
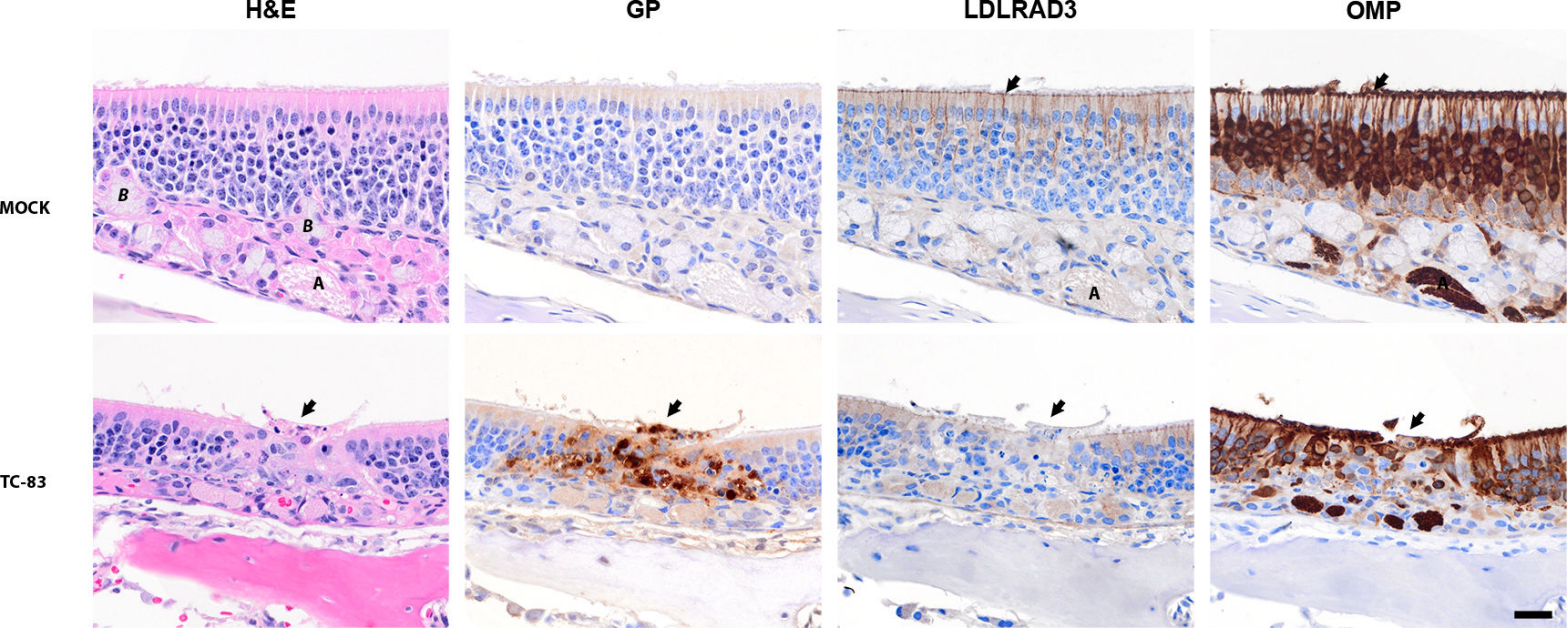
Representative images of the olfactory mucosa at 3 days post-infection with VEEV TC-83 in C3H/HeN mice. Representative serial images of the olfactory epithelium of 5- to 6-week-old, female, C3H/HeN mice infected with VEEV TC-83 (lower panels) or inoculated with PBS (upper panels). The staining used for each set of PBS or TC-83 infected tissues is shown above each panel. The far-left panel shows hematoxylin- and eosin-stained (H&E) tissue with an arrow pointing to destruction /necrosis in the olfactory epithelium for TC-83. Pyknosis and karyorrhexis can be seen near the epithelial layer. Mock-inoculated tissues stained with have an intact lining of pseudostratified cells in olfactory epithelium. The adjacent serial section shows IHC staining with the anti-VEE glycoprotein (GP) antibody counterstained with hematoxylin. The GP antigen was present in lesions in the TC-83-infected epithelial cells (center, arrow). The third panel from the left shows the distribution of the LDLRAD3 receptor by IHC. The LDLRAD3 signal was reduced in infected olfactory epithelium (see center, arrow) compared to surrounding no infected epithelium. In LDLRAD3, IHC of mock-inoculated epithelium, dense signals present in apical to middle part of the olfactory epithelium and OMP in adjacent sections. In the final panel, OMP in the mock-inoculated mice was observed in the cilia (arrow), dendrites, soma, and axon bundles (A—lamina propria) of olfactory neurons. In TC-83-infected epithelium, the OMP was reduced in the olfactory neurons in infected area (center arrow) compared to surrounding epithelium. Labels: Bowman’s gland (**B**), and axon bundles of olfactory neurons (**A**) located in the lamina propria. Scale bar = 20 µm.

**Fig 3:**
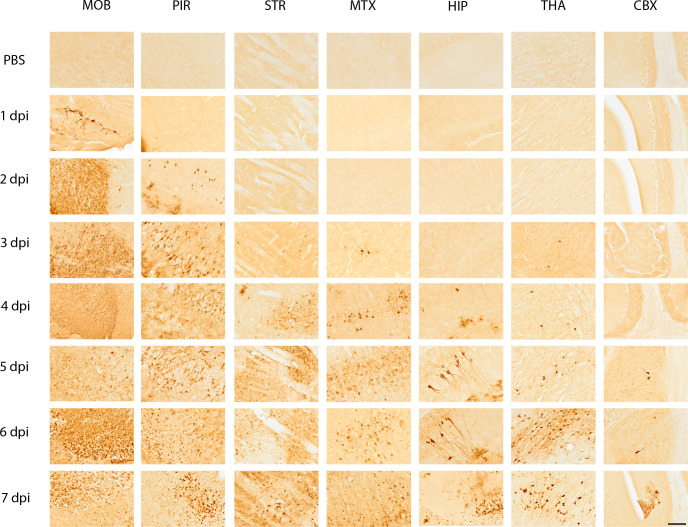
Representative immunohistochemical images of seven areas of the mouse brain probed for the presence of VEEV TC-83 glycoprotein (GP) from 1- to 7 days post-infection (dpi) and mock inoculated. Five- to six-week-old, female, C3H/HeN mice were inoculated intranasally with PBS (*n* = 4/day) or 10^7^ PFU of VEEV TC-83 (*n* = 4/day) and were sacrificed humanely on 1, 2, 3, 4, 5, 6, and 7 dpi. The mock-inoculated tissues are shown in the first row of the figure for each section of the brain. Each area of the brain is labeled at the top of the figure and the day post-infection is shown to the left of the figure. Seven regions of the brain are presented in the images: the MOB—main olfactory bulb; PIR—piriform cortex; STR—striatum; MTX—motor cortex; HIP—hippocampus; THA—thalamus; CBX—cerebellum. Images are at 20× magnification. VEEV GP was detected by immunohistochemistry of brains from mice, which is indicated by a dark brown color in the images. Images were chosen from sagittal sections that were cut with a Leica CM3050 at 40 µM thickness. Scale bar = 50 µm.

To further probe the dynamics of viral dissemination, we assessed the antigen distribution and associated pathology in the brain. C3H/HeN mice were intranasally inoculated with 10^7^ PFU of VEEV TC-83 or PBS (mock) on day 0, and then sacrificed to evaluate infection of the nasal epithelium (Fig. 2) and brain (Fig. 3). IHC staining of sections probed for the presence of VEEV TC-83 using antibody targeting the viral GP from 1 to 7 dpi. Sections of the brain were examined at the main olfactory bulb, piriform cortex, striatum, motor cortex, thalamus, hippocampus, and cerebellum for the presence of viral GP (Fig. 3).

The top layer of the olfactory epithelium was disrupted at sites of infection suggesting desquamation and cell death (Fig. 2, see H&E panel). In the nasal epithelium, viral GP antigen was readily detected in the olfactory sensory neurons, and the basal cells in the olfactory epithelium (Fig. 2, see GP panel). Beneath the olfactory epithelium in the lamina propria, GP antigen staining indicated infection in the axon of the olfactory neuron, and Bowman’s glands (Fig. S3). Infected cells show evidence of cell death as evidenced by pyknosis and karyorrhexis, i.e., cell nuclei that are condensed and fragmented (Fig. 2). IHC of the LDLRAD3 receptor showed widespread staining of the olfactory epithelium and staining suggested the receptor was present in the olfactory neurons (Fig. 2). Inspection of the lymphatic tissues in the nasal turbinates revealed scattered viral antigen within lymphatic tissues (Fig. S4B), colocalized with cells with dense or broken nuclear content (Fig. S4BC, see arrow). There was no notable staining of the LDLRAD3 in cellular content of lymphatic tissues in adjacent sections (Fig. S4C). Finally, we show the olfactory epithelium stained with OMP, a marker for mature olfactory receptor neurons (Fig. 2, see OMP panel). Comparison of OMP with the GP panels highlights the loss of the olfactory neurons at sites of infection.

**Fig 4:**
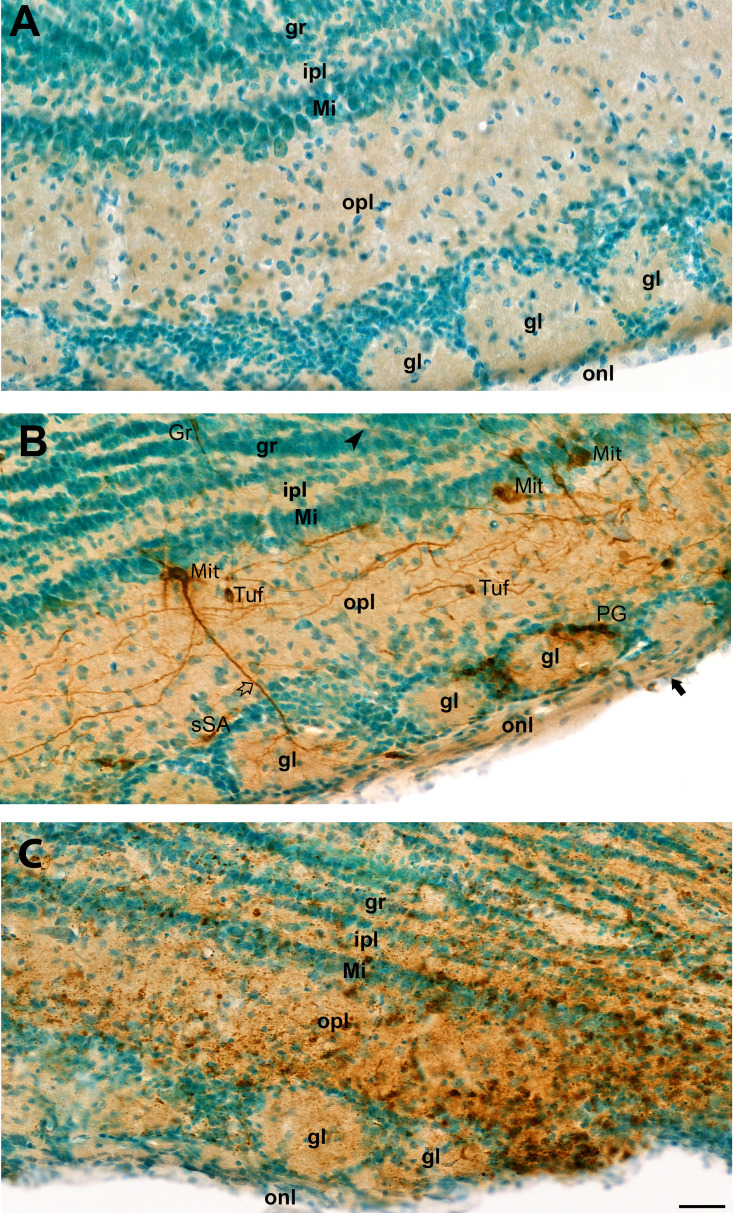
Representative images of olfactory bulb from mock or VEEV TC-83-inoculated mice showing glycoprotein (GP) antigen distribution. Brain tissue sections from mice were probed for VEEV TC-83 GP (brown color), and tissues were counterstained with methyl green. (**A**) Olfactory bulb from a mock-inoculated mouse showing normal anatomical structure with Olfactory nerve layer (onl), Glomerular layer (gl), Outer plexiform layer (opl), Mitral layer (Mi), Inner plexiform layer (Ipl), and Granule layer (gr). No staining for VEEV GP was evident in sham-inoculated specimens. (**B**) Representative image of the olfactory bulb after infection with VEEV TC-83 stained on 2 dpi. Punctuated labeling of GP was noted in axon in olfactory nerve layer (black arrow) with infected periglomerular (PG) cells around glomera and superficial short-axon (sSA) cells. GP is present in mitral cells (Mit) with punctuate in soma, dendrites (white arrow) in glomera, and axon (black arrowhead) in granular layer, Tufted cells (Tuf), and granular cells (Gr). (**C**) Mouse olfactory bulb from mouse infected with VEEV TC-83 at 6 dpi. The normal neuronal cellular features of soma, axon, and dendrites were no longer apparent in stained neurons, representing cellular damage from VEEV TC-83 infection. Tissue structures within each layer were mostly preserved. All three images have the same magnification. Scale bar = 40 µm.

In the main olfactory bulb, viral GP antigen was detected by 1 dpi in the dendrites and axons of neurons in each of the cell layers: olfactory nerve layer, glomerular layer, outer plexiform layer, mitral layer, inner plexiform layer, granule layer, and subependymal zone (Fig. 3). Tissue damage began at 2 dpi (Fig. 4B), after which virus disseminated throughout all neuronal cell types in the olfactory bulb (Fig. 4C). Despite widespread neuronal cell death by 6 dpi, the structural layers of the main olfactory bulb were intact (Fig. 4C).

**Fig 5:**
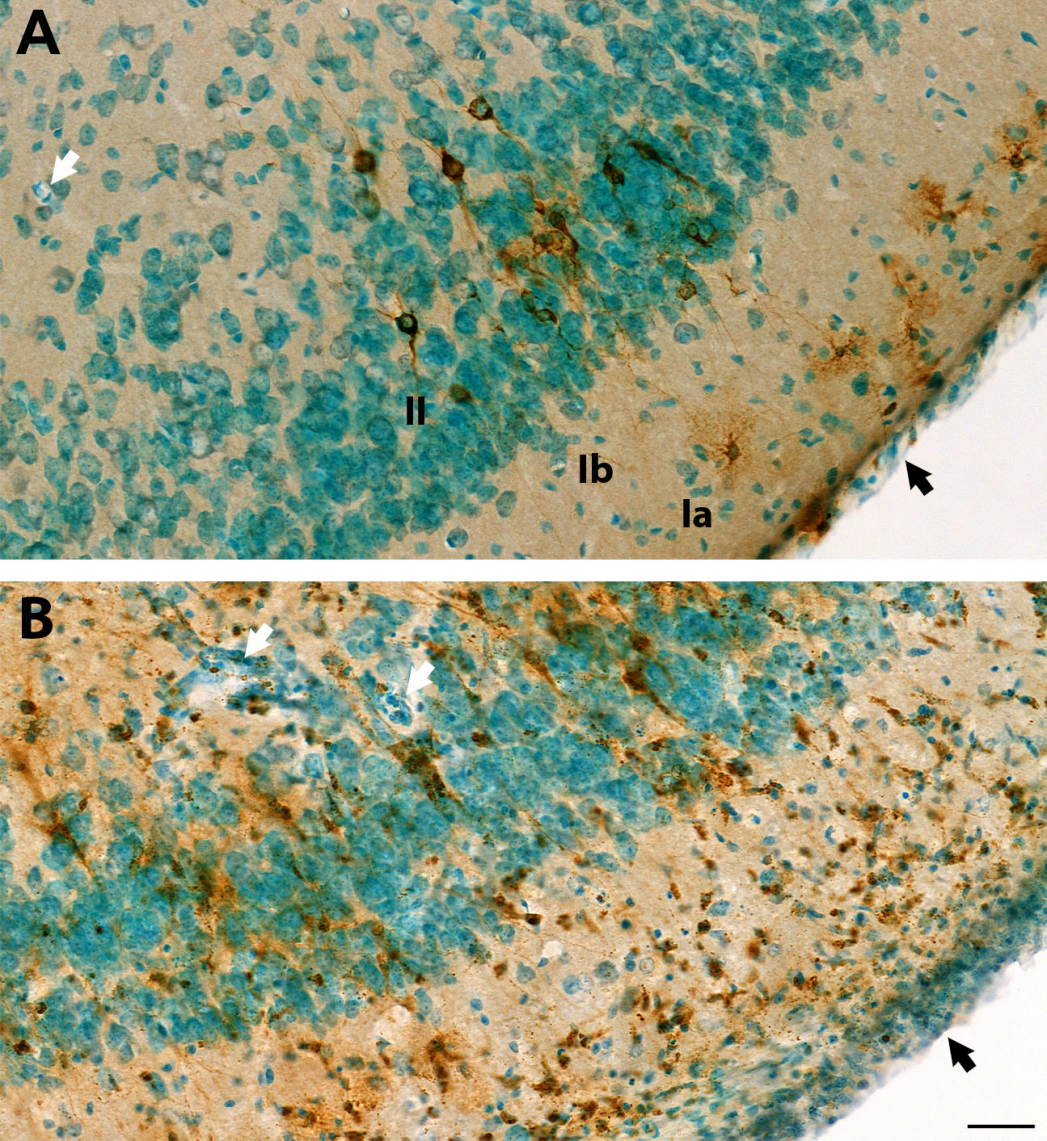
Additional images of VEEV-TC83 infection of the piriform cortex at 6 days post-infection. Additional images from the study presented in Fig. 3 to highlight damage in piriform cortex. Brain tissue sections of the piriform cortex region were probed for VEEV TC-83 glycoprotein (GP) by immunohistochemistry (IHC) (brown color) and slides of tissue sections were counterstained with methyl green. (**A**) At 2 dpi, IHC shows GP distributed in infected small pyramidal cell (brown color) in layer II (II) and several (morphological resemble to) neurogliaform neurons in the superficial part of layer I (Ia). Punctuated staining is present in soma and processes of infected neuron. The neuronal structure of VEEV TC-83-infected neurons and anatomical layer were well preserved. Blood vessels (white arrow) with thin walls, and thin meninges (black arrow) can be seen. Generally, at 2 dpi, neurons showed infection, but no marked damage or immune cell infiltration. (**B**) At 6 dpi, VEEV TC-83 infected neurons and cell debris were detected in all layers and cells with pyknotic nucleus were visible. Some cells showed small dense nuclear or clustered nonneuronal cells in all the piriform cortex layers. The wall of blood vessels (white arrow) and meninges (black arrow) are thicker and with more cell components and dense nuclear staining. Images in (A) and (B) have the same magnification. Scale bar = 40 µm.

By 2 dpi, viral GP was detected in the piriform cortex and increased thereafter (Fig. 3). Tissue damage in the piriform cortex began on 3 dpi and escalated in patches in successive time points (Fig. 3 and 5). A comparison of 2–6 dpi shows increased inflammation of the meninges and perivascular cuffing of 6 dpi (Fig. 5B versus C ). At 3 dpi, viral GP was observed in the striatum suggesting the virus moved from the piriform cortex into the striatal neurons in coronal sections of the ventromedial striatum (data not shown). Tissue damage in the striatum was greatest with microscopic lesions appearing at 4 dpi (Fig. 3). Individually infected neurons were seen in the cerebral cortex at 3 and 4 dpi, after which more widespread tissue damage occurred (Fig. 3).

**Fig 6:**
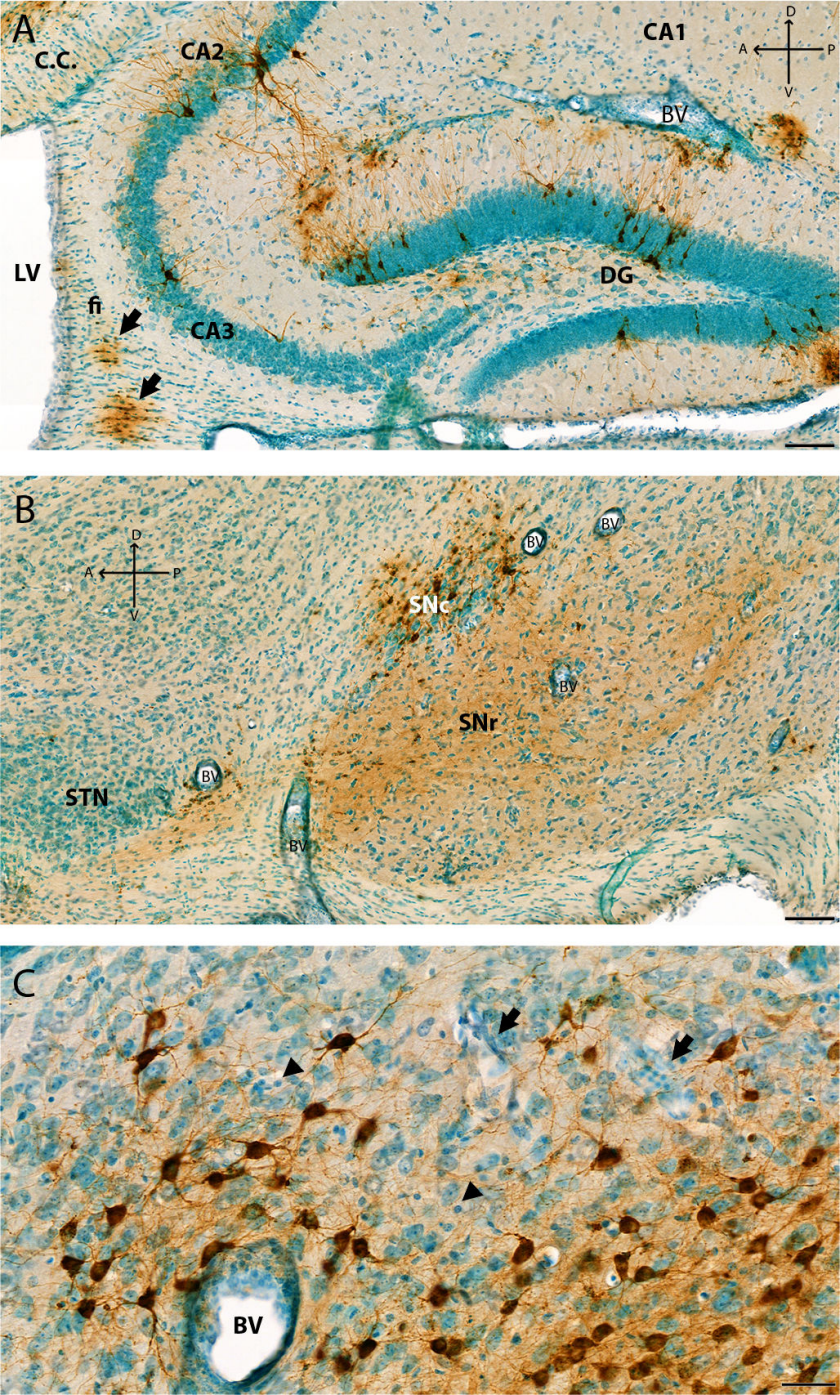
Additional images of VEEV-TC83 infection of the hippocampus, SNc, and thalamus at 6 dpi. Additional images from the study presented in Fig. 3 to highlight different neurons infected in the brain. (**A**) Image of the hippocampus at 6 dpi that shows VEEV TC-83-infected pyramidal cells in pyramidal layer, neurons in stratum lacunosum-moleculae layer, and neurons in granule layer: molecular layer and polymorph layer of dentate gyrus. Arrow shows infected oligodendrocytes in corpus callosum and fiber tracts. (**B**) IHC image of the midbrain shows soma of dopaminergic neurons in SNc and neuronal process in SNr. Thickened blood vessels walls (i.e., perivascular cuffing) are shown in panels (A) and (B) (arrow). Neuronal death is mild at 6 dpi in this region of the brain. (**C**) Representative IHC image of VEEV-TC83 infection of the thalamus nuclear at 6 dpi. VEEV-GP shown in brown color by IHC, and tissues were counterstained with methyl green. At 6 dpi, punctate GP signals were visible in soma and dendrites of thalamic neurons (brown) in mediodorsal thalamic nucleus, non-labeled neurons with rich cytoplasm and large nuclear with one or two nuclei. Increased clusters of cells were noted with small dense round nuclear (arrowhead). These cells were also seen in thickened walls of large blood vessels (BVs) and small blood vessels (arrow) potentially from immune cell infiltration and microglia activation. Pyknotic nuclei were observed suggesting infection and/or immune reaction, and cell death. Scale bar = 40 µm. CA1, Field CA1; CA2, Field CA2; CA3, Field CA3; CC, corpus callosum; CTX, cerebral cortex; DG, dentate gyrus; fi, fimbria of the hippocampus; mc, molecular layer; po, polymorph layer; sg, granule cell layer; slm, stratum lacunosum-moleculae; so, stratum oriens; sp, pyramidal layer; sr, stratum radiatum; SNc, substantia nigra pars compacta; SNr, substantia nigra pars reticulata; STN, subthalamic nucleus; VL, lateral ventricle. Scale bars: (A) and (B) = 100 µm; (C) = 40 µm; Cross: A: anterior; P: posterior: D: dorsal; V: ventral.

Viral GP antigen was detected in the hippocampus by 4 dpi, and finally, in a few dispersed neurons in cerebellum at 5 dpi (Fig. 3). In the hippocampus, lesions began by 5 dpi, within the granule cell layer of dentate gyrus, where neurons connecting the outer and inner layers can be seen (Fig. 6A). Infection appeared to begin within the CA2 (cornu ammonis) region within the hippocampus (Fig. 6A). GP antigen was noted in the white matter/corpus callosum/fiber tracts in oligodendrocytes (Fig. 6A). In the basal ganglia, we noted stained neurons with large soma and dendrites morphology resemble to dopaminergic neurons in the Substantia nigra compacta (SNc) and dendrites extended to the Substantia nigra reticulata (SNr) (Fig. 6B). Staining was observed in neuronal fibers. Individual mossy fiber neurons connecting the dentate gyrus to the pyramidal cell layer showed GP antigen staining at 5 dpi (Fig. 3). VEEV TC-83 reached the thalamus by 5 dpi and viral antigen levels increased by 6 and 7 dpi when greater numbers of cells were infected (Fig. 3). Intensive staining of the neuronal soma and process of the thalamic neurons was evident with perivascular cuffing and influx of numerous inflammatory cells (Fig. 6C). Within the cerebellum, individual Purkinje cells were infected by 7 dpi and neuronal death was apparent by morphology (Fig. 3).

### Lymphocytes, monocytes, and macrophage populations increased through 7 dpi, while microglia populations decreased indicating cell death

**Fig 7:**
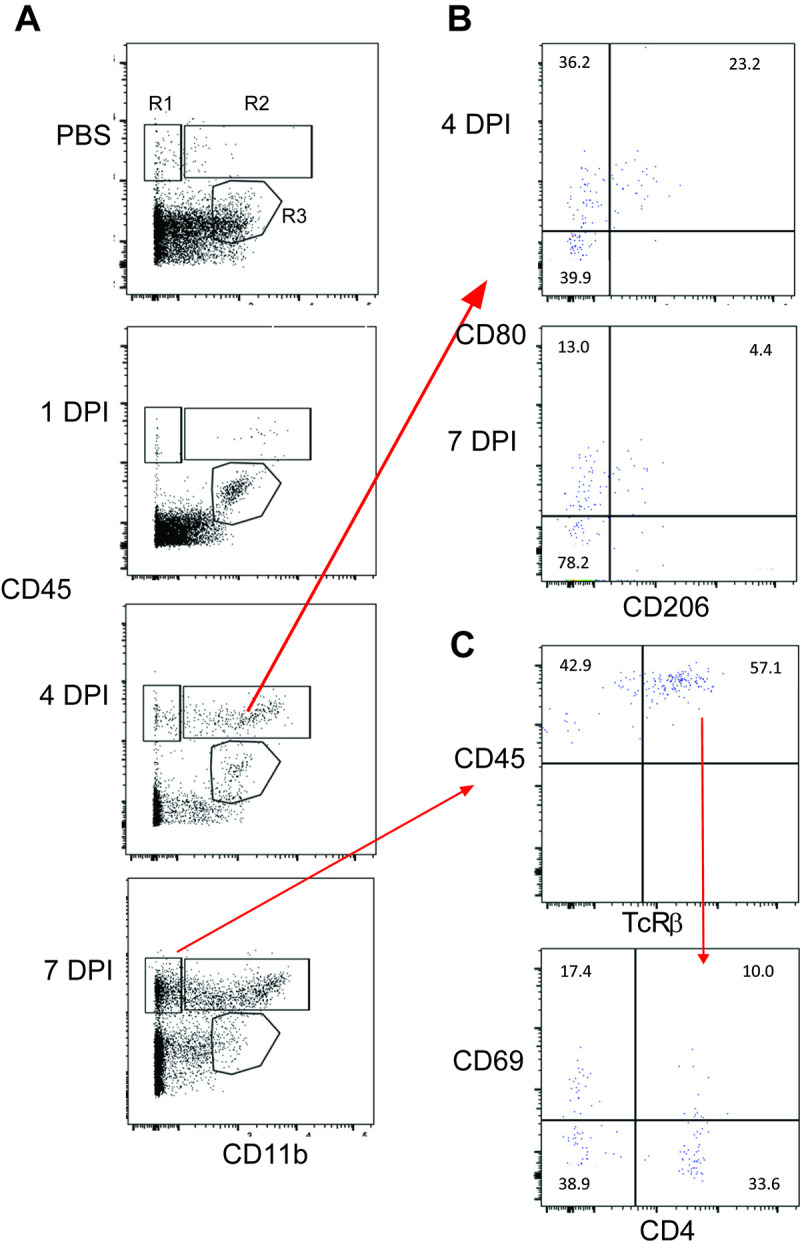
Kinetics of immune cell infiltration in C3H/HeN mouse brains following VEEV TC-83 infection. Cells were isolated from the brains of mice infected with a lethal dose of VEEV TC-83 and sacrificed at 1, 4, and 7 dpi and analyzed by flow cytometry. (**A**) Representative flow cytometry plots demonstrating the infiltration of immune cells over time based on CD45 and CD11b expression. R1 gate (CD45^hi^ CD11b^−^) includes lymphocytes, R2 gate (CD45^hi^ CD11b^+^) includes monocytes and macrophages and the R3 gate (CD45^int^ CD11b^+^) includes the microglia population. (**B**) Representative flow cytometry plots demonstrating changes in CD80 and CD206 expression on myeloid cells in R2 gate from 4 to 7 dpi. (C) Representative flow cytometry plot demonstrating increased expression of CD69 on T cells (TcRβ^+^).

To identify changes in the immune cell populations in the brains of TC-83 mice as viral infection progressed, we performed flow cytometry on brain cells isolated from infected mice sacrificed on 1, 4, and 7 dpi (Fig. 7). Analysis of cell surface staining with antibodies to CD45 and CD11b reveal three populations of immune cells in the brain, cells that were CD45^hi^ CD11b^lo^ (R1 gate) which includes lymphocytes, cells that were CD45^hi^ CD11b^hi^ (R2 gate) which contains myeloid cells, such as monocytes, macrophages, DCs, and neutrophils, and finally, the cells that were CD45^int^ CD11b^+^ gate (R3 gate) which includes microglia (Fig. 7A). At 1 dpi, the immune cell profile in TC-83 infected mice was similar to uninfected control mice, whereas by 4 dpi, mice infected with TC-83 exhibit increases in lymphocytes and monocytes/macrophages. These populations continued to increase through 7 dpi. Conversely, there was a decrease in cells in the R3 gate which began at approximately 4 dpi and continued to decrease until 7 dpi suggesting the loss of these cells.

The phenotype of the monocyte/macrophage cells (R2 population) that infiltrated the brain revealed three subpopulations that changed in frequency from 4 to 7 dpi (Fig. 7B). CD80 is a pro-inflammatory marker and single positive CD80 cells made up 28.5 ± 5.7% of R2 cells on 4 dpi and decreased to 16.5 ± 3.6% by 7 dpi. Double positive CD80^+^ CD206^+^ cells made up 22.0 ± 2.8% of R2 cells at 4 dpi and decreased to 3.9 ± 1.3% by 7 dpi. Conversely, the double negative cells in this gate increased from 48.5 ± 7.8% at 4 dpi to 75.8 ± 1.9% at 7 dpi.

In addition to myeloid cells increasing in the brains of TC-83 mice, there was also an increase in lymphoid cells by 7 dpi (Fig. 7C). Classical αβ T cells made up approximately 56 ± 2.3% of the cells in the R1 gate, of which 43 ± 6.4% are CD4^+^ T cells. Although a small percentage of the CD4^+^ T cells were activated as measured by CD69 expression (14 ± 3.3%), the majority of the CD69^+^ T cells were CD4^−^ (37 ± 15%).

**Fig 8:**
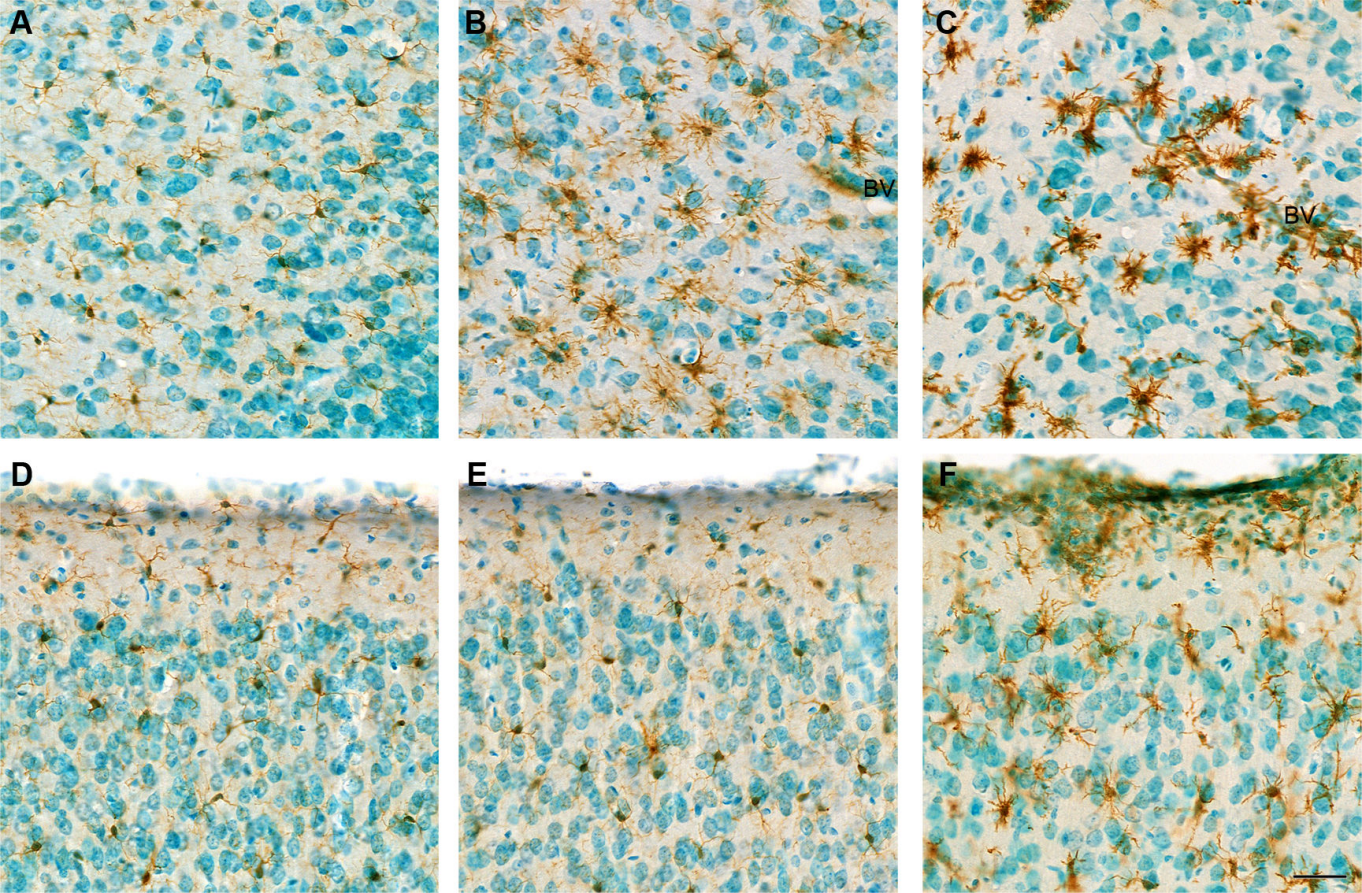
Representative images of microglia in the piriform and motor cortex from mock and VEEV TC-83-inoculated mice. Images from the piriform cortex (**A**) and motor cortex (**D**) are shown from a mock-inoculated mouse brain. Similarly, piriform cortex (**B, C**) and motor cortex (**E, F**) sections are presented from TC-83 infected mouse brains. Sagittal brain sections were stained by immunohistochemistry with the microglia marker Iba-1 and counterstained with methyl green from tissues taken at 2 dpi (**A, B, D, E**) and 6 dpi (**C, F**). Microglia were present with fine, long processes with round or elongated cell nuclear distributed. At 2 dpi, microglia with more processes were seen in the piriform cortex of TC-83 infected mice with some pyknotic nuclear and thickened wall of blood vessels (BVs). At 6 dpi, microglia were thicker and shorter with some clustered around damaged neurons and thickened blood vessels wall (**C, F**). Increased numbers of cells with dense nuclear not stained with Iba-1 that may be infiltrating immune cells. Pyknosis was apparent in some cells. All images have the same magnification. Scale bar = 40 µm.

**Fig 9:**
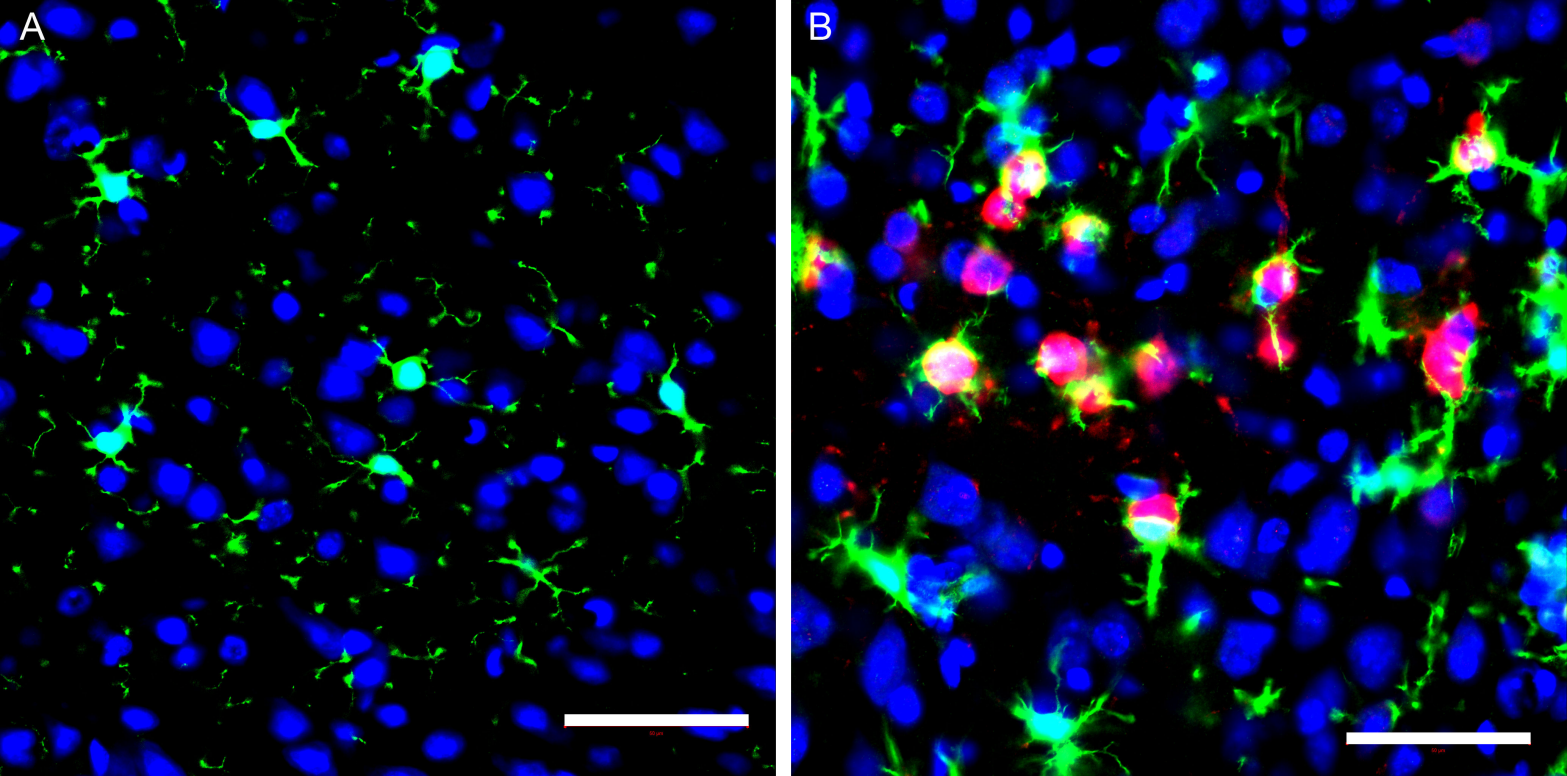
Representative immunofluorescent images of the microglia and virus in the motor cortex in mock and VEEV TC-83 inoculated mice at 6 dpi. Sagittal sections of the brain were stained with Iba-1 (green), VEEV TC-83 (red), and nuclei (blue). (**A**) Representative image of motor cortex shows layers I and II from a mock-inoculated mouse brain. Microglia were present with fine, long processes with round or elongated cell nuclear distributed in brain tissues. (**B**) Representative image of motor cortex shows soma and processes of infected neurons (red) near microglia cells. Activated microglia were thick with short process. Scale bar = 50 µm.

Evaluation of microglial cells by IHC in the piriform and motor cortex showed a morphology with thicker and shorter processes following infection (Fig. 8). Microglial cells were aggregated around neuronal cells (Fig. 8B, C, E and F) as compared to mock-inoculated (PBS) mice (Fig. 8A and D). Double-staining of the VEEV antigen and microglial cells at 6 dpi show microglial cells wrapped around viral-infected neurons (Fig. 9).

### Deep spatial RNA-Seq profiling reveals a strong pro-inflammatory response and cell death

To study the spatial and dynamic response of the host response of the brain to VEEV TC-83 infection, we used the RNA-Seq data from 1, 3, 5, 6, and 7 dpi for each of the eight areas of the brain from virus and mock-inoculated mice. Analysis of the differential gene expression was assessed by comparison of brain regions from infected mice from 1, 3, 5, 6, or 7 dpi to mock-inoculated mice (Table 1). At 1 dpi, the main olfactory bulb had the highest number of differentially expressed genes compared to the other brain areas by more than a factor of 2, with the remaining seven areas having a similar number of differentially expressed genes. By 3 dpi, the number of differentially expressed genes increased in all areas except for that within the thalamus and cerebellum which saw a decrease. At 5 dpi, the number of differentially expressed genes in each area increased further compared to 3 dpi except for within the main olfactory bulb. By 6 and 7 dpi, the number of differentially expressed genes increased in all areas.

**TABLE 1:** Number^*a*^ of differentially expressed genes in eight regions of the brain

	Days post-infection				
Brain Area	1	3	5	6	7
MOB	1,689	1,977	1,786	2,285	2,726
PIR	624	981	1,400	2,327	2,424
STR	599	790	1,343	1,935	2,081
MTX	558	688	1,397	1,989	2,352
HIP	540	670	1,324	1,873	2,318
STX	563	642	1,542	1,945	2,257
THA	524	495	985	1,852	1,797
CBX	580	509	669	1,079	1,380

^a^ Number of differentially expressed genes in each portion of the brain (Log_2_ fold change ≤2 and ≥2; false discovery rate ≤0.05). Abbreviations: CBX, cerebellum; HIP, hippocampus; MOB, main olfactory bulb; MTX, motor cortex; PIR, piriform cortex; STR, striatum; STX, sensory cortex; THA, thalamus.

**Fig 10:**
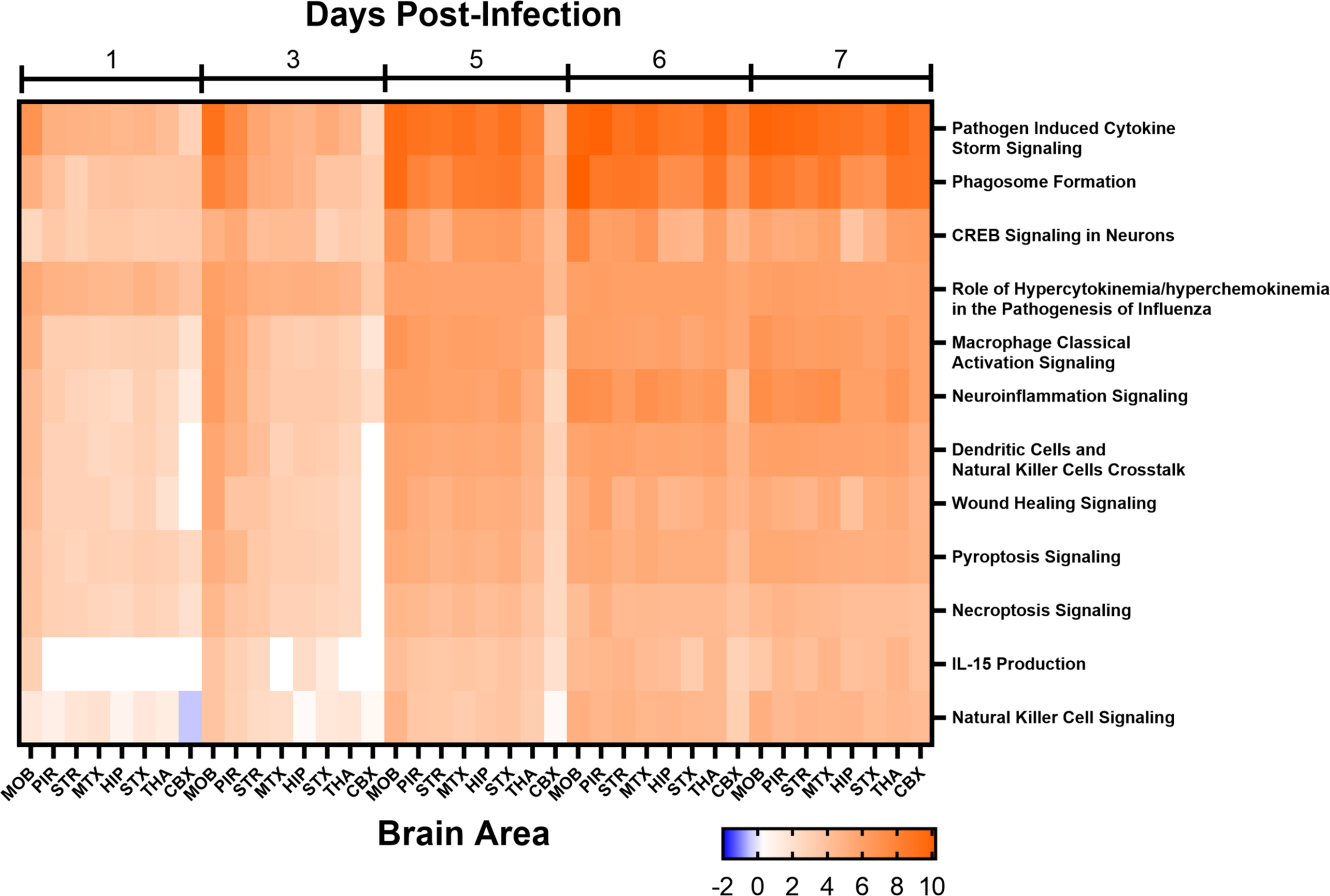
Major activated canonical pathways in eight areas of the brain from mice intranasally (i.n.) infected with VEEV TC-83 at 1, 3, 5, 6, and 7 days post-infection (dpi). Mice (*n* = 4) were i.n. infected with VEEV TC-83 or mock inoculate with PBS. At 1, 3, 5, 6, and 7 dpi brains were harvested from VEEV TC-83-infected mice and at 1 and 5 dpi brains were harvested from mock-infected mice for RNA-Seq. Differential gene expression was calculated using DESeq2 for each day of the VEEV TC-83-infected samples using all of the mock-infected mice as controls. Differentially expressed genes (Log_2_ fold change ≤−2 and ≥2; false discovery rate ≤0.05) were used to assess canonical pathways activation using Ingenuity Pathway Analyses. The activation score is noted in the color bar below the figure. Abbreviations: CBX, cerebellum; HIP, hippocampus; MOB, main olfactory bulb; MTX, motor cortex; PIR, piriform cortex; STR, striatum; STX, sensory cortex; THA, thalamus.

The canonical activated pathways were defined based on the DESeq2 results using Ingenuity Pathway Analysis for 1, 3, 5, 6, and 7 dpi, which compared infected to mock-inoculated mice. The most significantly activated pathways associated with infection were pathogen-induced cytokine storm signaling pathway, phagosome formation, CREB signaling in neurons, role of hypercytokinemia/hyperchemokinemia in the pathogenesis of influenza, and macrophage classical activation signaling pathway (Fig. 10). Several activated pathways were associated with immune cell activation such as macrophages and natural killer cells. The most extensive network of genes upregulated was noted in the neuroinflammatory signaling pathway. In general, pathway activation increased for all sites within the brain from 1 to 7 dpi.

**Fig 11:**
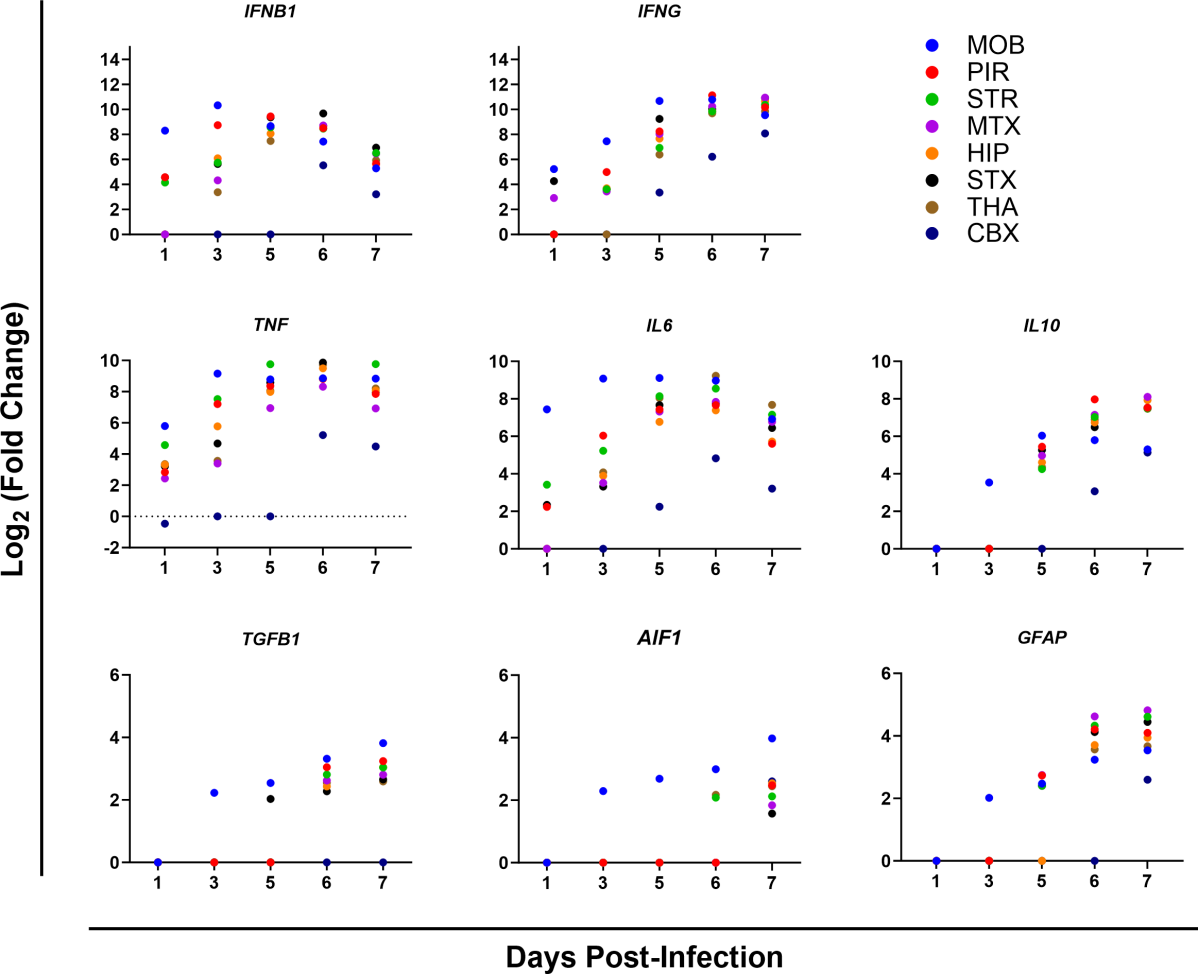
Upregulation of selected genes from major canonical pathways in eight areas of the brain from mice intranasally (i.n.) infected with VEEV TC-83 at 1, 3, 5, 6, and 7 days post-infection (dpi). Mice (*n* = 4) were i.n. infected with VEEV TC-83 or mock inoculated and processed by RNA-Seq. Differential gene expression was calculated using DESeq2 for each day of the VEEV TC-83-infected samples using all of the mock-inoculated mice as controls. All Log_2_ fold change values are statistically significant. Abbreviations: CBX, cerebellum; HIP, hippocampus; MOB, main olfactory bulb; MTX, motor cortex; PIR, piriform cortex; STR, striatum; STX, sensory cortex; THA, thalamus.

**Fig 12:**
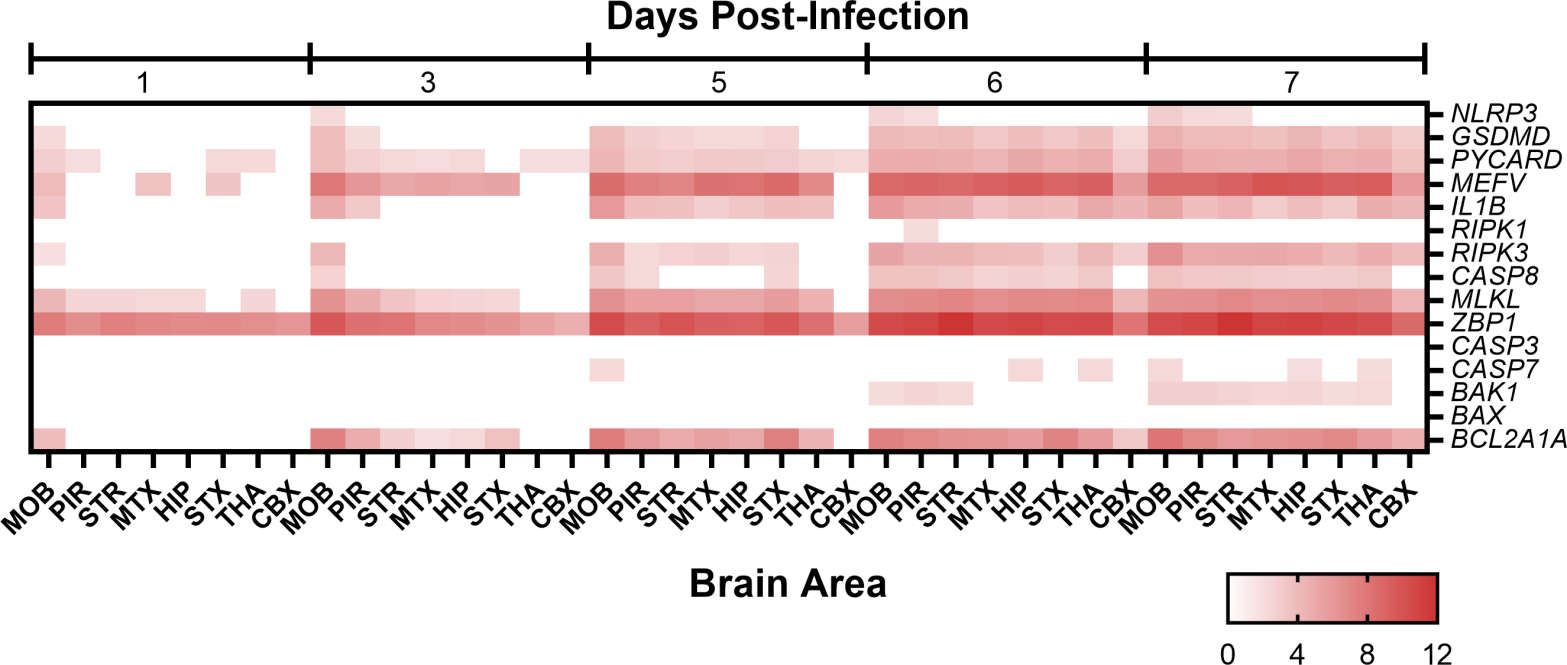
Upregulation of select genes from programmed cell death pathways in eight areas of the brain from mice intranasally (i.n.) infected with VEEV TC-83 at 1, 3, 5, 6, and 7 days post-infection (dpi). Mice (*n* = 4) were i.n. infected with VEEV TC-83 or mock inoculated. At 1, 3, 5, 6, and 7 dpi brains were harvested from VEEV TC-83-infected mice and at 1 and 5 dpi brains were harvested from mock-inoculated mice for RNA-Seq. Differential gene expression was calculated using DESeq2 for each day of the VEEV TC-83-infected samples using all of the mock-inoculated mice as controls. The heatmap shows the log_2_ (fold change) of genes from three major cell death pathways: pyroptosis (*NLRP3*, *GSDMD*, *PYCARD*, *MEFV*, and *IL1B*), necroptosis (*RIPK1*, *RIPK3*, *CASP8*, *MLKL*, and *ZBP1*), and apoptosis (*CASP3*, *CASP7*, *BAK1*, *BAX*, and *BCL2A1A*). The log_2_ fold change increase is noted in the color bar below the figure. All values are statistically significant. Abbreviations: CBX, cerebellum; HIP, hippocampus; MOB, main olfactory bulb; MTX, motor cortex; PIR, piriform cortex; STR, striatum; STX, sensory cortex; THA, thalamus.

Analysis of major genes in highly activated pathways revealed strong pro-inflammatory responses with the upregulation of the *IFNB1*, *IFNG*, *TNF*, *IL10*, and *IL6* (Fig. 11). Within the pyroptosis pathway, *NLRP3* levels were observed to be at baseline levels and were only noted in a small number of brain areas at 6 and 7 dpi, whereas genes downstream of it, such as *GSDMD*, *PYCARD*, *MEFV*, and *IL1B* exhibited increased expression at 5 dpi with the number of brain areas with high levels being more as compared to *NLRP3* (Fig. 12). In the necroptosis pathway, *RIPK3* had high expression levels in a majority of brain areas starting on 5 dpi, whereas *RIPK1* was only significantly expressed in one brain area at 6 dpi and *CASP8* expressed at elevated levels at 6 dpi in most brain areas. Other necroptosis associated the genes, *MLKL* and *ZBP1*, were significantly expressed with both genes exhibiting expression from 1 dpi, but only *ZBP1* expression being observed in each brain area sampled. Finally, the programmed cell death pathway, apoptosis signaling pathway saw limited activation with baseline expression of *CASP3* and *CASP7* expression only observed a limited number of times. Similarly, the proapoptotic genes *BAX* and *BAK1* exhibited limited expression, whereas the antiapoptotic gene, *BCL2A1A* saw increased expression as early as 3 dpi in a majority of brain areas and levels increased through 7 dpi.

In addition to the host responses attributed to microglial activation and neuronal death described above and in the prior section, astrocytes responded to infection as noted by the elevation of GFAP (Fig. 12). Staining of GFAP in the piriform cortex, striatum, and entorhinal cortex suggest increased numbers of astrocytes by 6 dpi (representative image of piriform cortex in Fig. S5).

## DISCUSSION

Apart from acyclovir for treating herpes simplex virus infections of the CNS, we have no direct-acting antiviral drugs for treatment of neurotropic viral infections. Discovery and development of these types of drugs present certain challenges. The therapeutic treatment of viral encephalitis requires drugs that pass the blood-brain barrier, are safe, provide ample exposure to thwart viral replication and enable the immune system to eliminate the threat. Drug treatment regimens should also mitigate the potential for the selection of resistance of the virus and damage from the host response to infection. Moreover, we must understand how the timing and dose of treatment might impact biodistribution and how this in turn may impact the effectiveness of the drug. Hence, in addition to standard drug pharmacokinetic, safety, and efficacy studies, the evaluation of therapeutics that target viral infections of the brain demands knowledge of the host’s response to the virus, dissemination, and location of the virus, and the potential for within-host evolution of the virus. To address these questions, we made a comprehensive evaluation of the infection, dissemination, and genetic variation of intranasal infection of VEEV TC-83 in the mouse, and additionally, we evaluated the dynamics of the host response. These studies reveal the potential challenges in treatment of VEEV infection over time and suggest potential approaches in the development and assessment of an antiviral for its treatment.

Human cases of VEE results in febrile disease with some patients exhibiting lymphopenia and/or neutropenia (3, 4). In less than 1% of cases, VEE progresses to neurological disease with edema, meningitis, and congestion from mononuclear inflammatory infiltrates (3 - 5). The most common clinical signs of infection with VEEV TC-83 in the mouse strain, C3H/HeN, include weight loss, hunching, alertness/lethargy, and mice eventually reach a moribund state requiring humane euthanasia at 7 dpi or greater depending on dose of infection (33, 36). One of the main clinical signs was that the mice were unresponsive to stimulation. This clinical observation may be explained by the extensive infection of the brain noted herein, and other reports cited herein, in the basal ganglia of the caudate putamen, substantia nigra, motor cortex, thalamus, and cerebellum, which participate in motor control and movement. The thalamus also assists in consciousness and alertness, while acting as a relay center for most sensory pathways, which may result in lethargy. Loss or damage of dopaminergic neurons in SNc due to infection could cause parkinsonism has been reported in human Western equine encephalitic cases (37, 38). A multiple sclerosis-like syndrome has also been proposed based on VEEV-mouse models that show demyelination of the spinal cord and proposed to be driven by the host immune response (39, 40).

Herein, we examined the distribution of viral antigen, viral RNA levels, and genetic variation of viral genomes over time in the mouse brain following infection with VEEV TC-83 along with the host’s response to infection. The RNA-Seq and the IHC analysis of VEEV TC-83-infected brains from C3H/HeN mice show that the virus reaches the olfactory bulbs and most other parts of the brain (albeit low) within 24 h after intranasal infection. Notably, there was a low level of genetic diversity at 1 dpi within the olfactory bulbs which was followed by an expansion of genetic diversity which peaked at 5 dpi. By 7 dpi, diversity fell. Viral RNA levels peaked in the main olfactory bulbs and piriform cortex by 3 dpi and decreased slightly thereafter through 7 dpi, but this did not correlate with genetic diversity. By 3 dpi, the piriform cortex shows signs of cellular damage, which continued to increase as noted by perivascular cuffing, and diffused meningitis. By 5 dpi, numerous areas show lesions, and RNA-Seq suggested a significant increase in the neuroinflammatory response. The rise in pathology and inflammation correlated with a dramatic rise in Ns mutations. Interestingly, by 7 dpi, the number of Ns mutations decreased in the olfactory bulbs, piriform cortex, thalamus, and hippocampus, but remained elevated in the motor cortex, striatum, and sensory cortex. This may reflect continued replication in motor cortex, striatum, and sensory cortex in contrast to other areas with fewer viable neurons and increased cell death. The distribution of the viral antigen matches well with published data on the distribution of viral antigen in this mouse model, in which the time points for the appearance of virus in the piriform cortex, caudate putamen, thalamus, and hippocampus were the same (33, 36). The most notable finding from these studies was the expansion of Ns mutations throughout the brain. Network analyses of the consensus sequences (based on a 50% majority or a nucleotide majority for more than one substitution) suggest that nucleotide changes give rise to distinct genetic variants in 9 of the 20 mice brains examined (Fig. S6). Importantly, these variants were spread throughout the brain in the individual; however, the nucleotide sequence of the variants each differed among the mice. In other words, each mouse had a distinct genetic variant (Fig. S6). The biological fitness and relevance of these genetic variants will be important to assess in future studies. These studies were limited by the number of mice evaluated on each day (*n* = 4) and future studies would benefit from additional mice to define the probability of occurrence of individual Ns mutations and noted consensus sequences.

There was a significant increase in immune cells in the brain by 4 dpi indicated by an increase in cells in the lymphocyte (R1) and monocyte/macrophage (R2) gates which correlates well with the kinetics of viral invasion of the brain and the RNA-Seq data. It has been suggested that CD4^+^ T cells are protective in this model (10, 24) and we observed an increase of CD4^+^ T cells in the brain by 7 dpi. However, only a small percentage of these cells were activated as measured by CD69 expression and they did not appear to offer protection because mice either succumbed to infection or had to be euthanized at this time point. Conversely, there was an increase in activated (CD69^+^) non-CD4^+^ T cells which are presumably cytotoxic CD8^+^ T cells. Increased cytotoxic activity against virally infected neurons may contribute to the cell death and pathology observed at the later time points; however, the role of CD8^+^ T cells in the disease process has not been firmly elucidated for any of the VEEV strains. It is likely that NK cells, B cells, or T cells make up the remainder of the cells in the lymphocyte gate. The increase in myeloid cells is also evident at 4 dpi and approximately half of the cells express the co-stimulatory marker CD80 which is typically expressed on DCs and macrophages and indicative of a pro-inflammatory state. This is consistent with the RNA-Seq data which demonstrated an increase in numerous pro-inflammatory cytokines, chemokines, and other inflammatory host response genes by 5 dpi. These data also confirm results from other with studies demonstrating an increase in pro-inflammatory cytokines such as TNF, IL-6, IL-1β, and IFN- in VEEV-infected mice (41 - 43). Interestingly, a small percentage of these cells also expressed CD206 which is a marker of alternative activation and is indicative of tissue repair and remodeling. However, by 7 dpi, there was a decrease in the CD80^+^ CD206^+^ cell population and a slight decrease in single positive CD80 cells, suggesting either a downregulation of the markers or a loss of those cells. Conversely, the cells within the R3 gate which includes the microglia population decreased from 1 dpi through 7 dpi which may reflect cell death and correlates with the IHC staining and RNA-Seq data demonstrating an increase in cell death markers. However, it is possible that the cells within the R3 gate altered their expression of CD45 as the dpi increased and moved out of this gate.

This model suggests three stages of infection and disease to consider during therapeutic treatment of brain infection, the early stage of infection (1–3 dpi), midway (4–5 dpi), and late (6–8 dpi). The earliest stages of infection are characterized by low levels of infectious virus and minimal inflammation. During this period, antivirals that attack the virus may well show the greatest efficacy and protection from pathogenesis. High levels of virus and inflammation characterize the timeframe midway during infection and will therefore require greater exposure of the drug in the brain. It is unknown if elimination of the virus during this stage will permit adequate healing and regeneration of damaged brain tissue. Treatment of the disease in the last stage which is characterized by widespread cell death, continued inflammation, and infectious virus represents the greatest challenge. The levels of drug exposure required may change again due to alterations of the BBB and deterioration of the endothelial cell network resulting in difficulty in reaching all areas of the brain. Future efforts will seek to assess efficacy and drug exposure in each of these stages with VEEV antivirals (34, 35).

## Data Availability

All raw sequence files and their associated processed files are publicly available under the Gene Expression Omnibus accession series GSE213725.

## SUPPLEMENTAL MATERIAL

Supplemental material jvi.00827-23-s0001.docx Supplemental figures and table.

ASM does not own the copyrights to Supplemental Material that may be linked to, or accessed through, an article. The authors have granted ASM a non-exclusive, world-wide license to publish the Supplemental Material files. Please contact the corresponding author directly for reuse.
