# Supplementary material for: Deep spatial profiling of Venezuelan equine encephalitis virus reveals increased genetic diversity amidst neuroinflammation and cell death during brain infection
Source: J Virol. 2023 Aug 10;97(8):e00827-23. doi: 10.1128/jvi.00827-23 (PMC10506382; doi:10.1128/jvi.00827-23)
Supplement: Supplemental material — Supplemental figures and table. [file jvi.00827-23-s0001.docx]

**Supplemental Figures and Table**

**
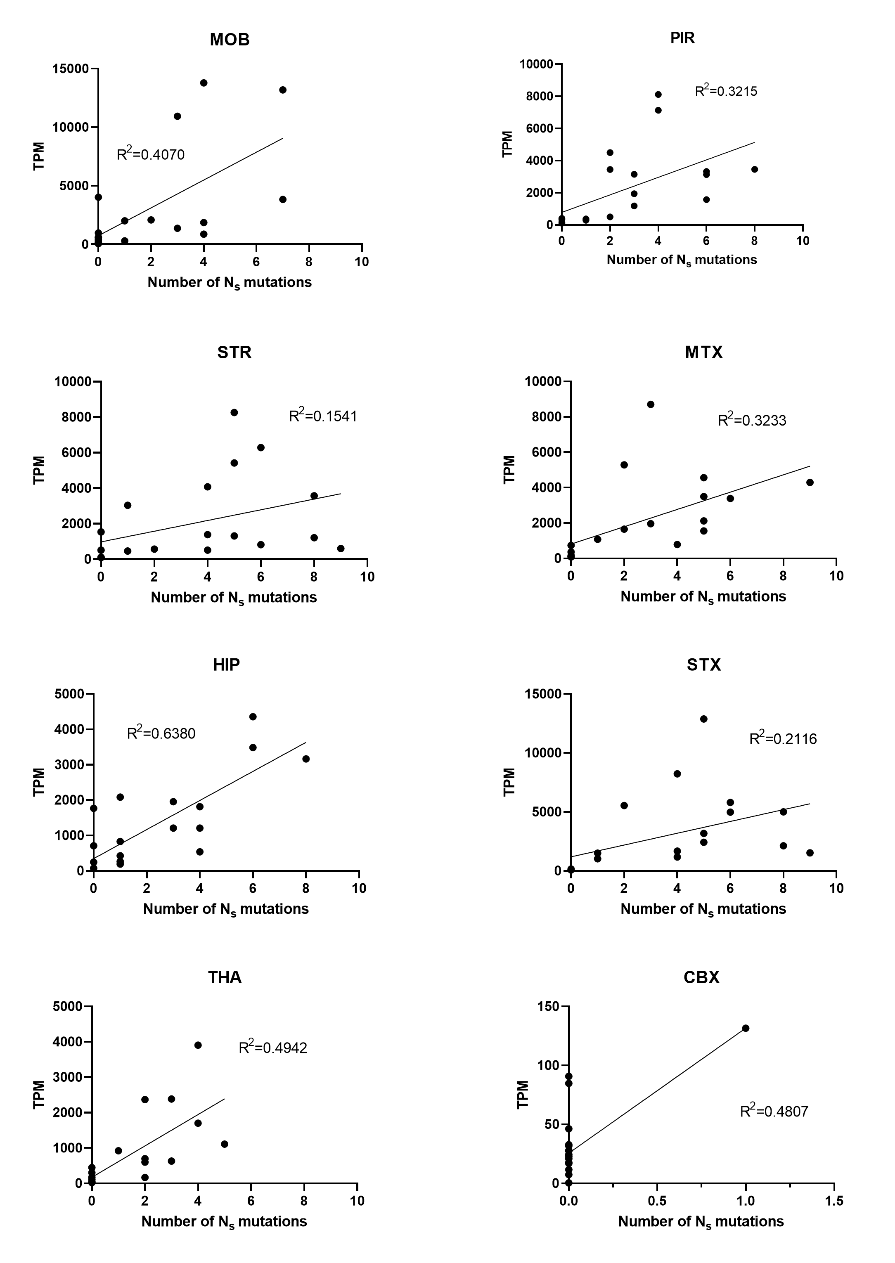
**

**Figure S1. Correlation between VEEV TC-83 genome transcripts per million and nonsynonymous mutations at 1-, 3-, 5-, 6-, and 7-days post-infection.** Mice (n=4/day) were intranasally infected with VEEV TC-83 and at 3-, 5-, 6- and 7-days post-infection, their brains were harvested and divided into eight portions and RNA-Seq performed on to determine transcripts per million and Ns mutations as described in materials and methods.

**
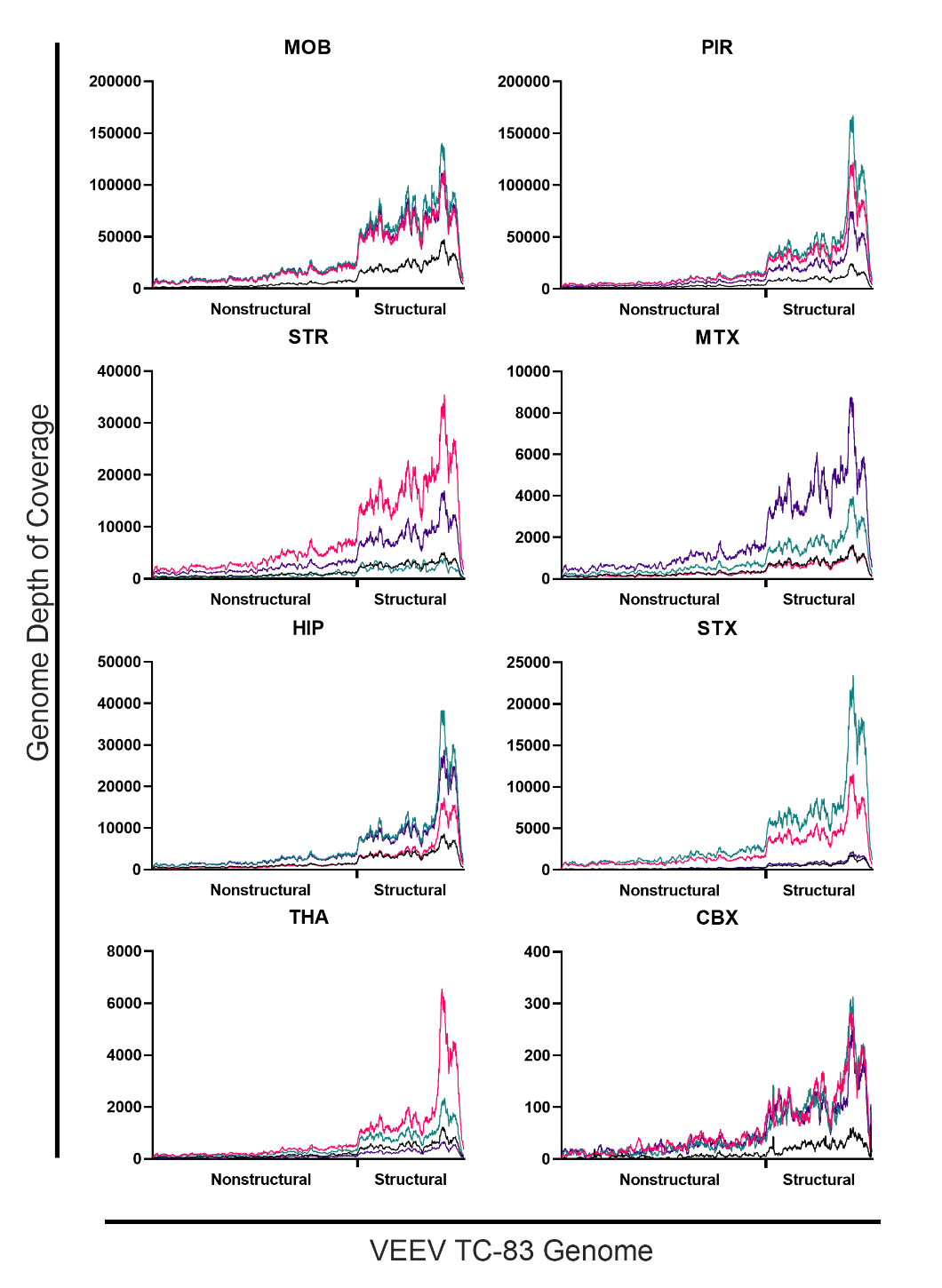
**

**Figure S2. Genome depth of coverage of VEEV TC-83 in brain areas of infected mice at 5-days post-infection.** Mice (n=4/day) were intranasally infected with VEEV TC-83 and 5-days post-infection, their brains were harvested and divided into eight portions and RNA-Seq performed on to determine depth of coverage.


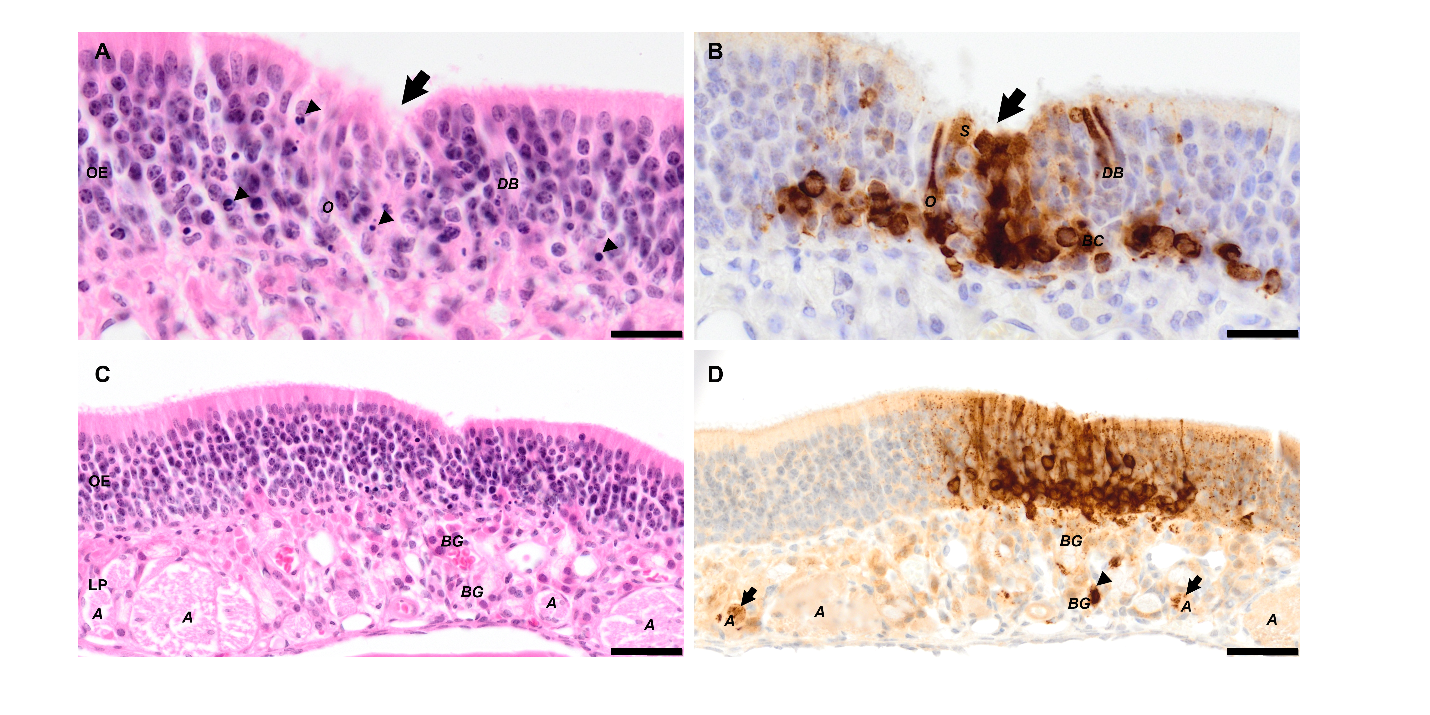


**Figure S3. H&E and glycoprotein staining of VEEV TC-83 infected olfactory mucosa at day one post infection.** Higher magnification of images of **(**A) H&E-stained section of the epithelial layer of the olfactory mucosa in an infected mouse on one day post infection. Image of H&E-stained olfactory epithelium shows focal tissues loss (large ***arrow***), inflammatory cells infiltration, pyknosis (small ***arrowhead***) in olfactory epithelium, some with morphological feature of olfactory epithelial cells ***OE***: olfactory epithelium; ***O***: olfactory neuron, ***DB***: duct of Bowmans’s gland. (**B**) An adjacent section showing staining of the glycoprotein (GP) by IHC. GP staining was noted in several different cell types in olfactory epithelium: ***O***: olfactory neurons; ***S***: supporting cells; ***BC***: Basal cells; ***DB***: duct of Bowmans’ gland. Panels C and D show images of olfactory mucosa at lower magnification. (C) H&E shows similar pattern as in higher mag. **OE**: olfactory epithelium; **LP**: *lamina propria*; ***BG***: Bowman’s gland; ***A***: representative axon bundles. (**D**) Adjacent section of VEE-GP IHC: Majority of signa was in dendrites (in apical layer), soma (in intermediate) of olfactory neurons and basal cells (in basal cell layer) in olfactory epithelium as well as Bowman’s gland (***BG***) and axon bundles (***A***) in lamina propria. ***Arrow head***: infected acinar cells of Bowman’s gland; and infected axon bundles (***Arrow***). Scale bar: A and B=20 µm; C and D=40 µm

**
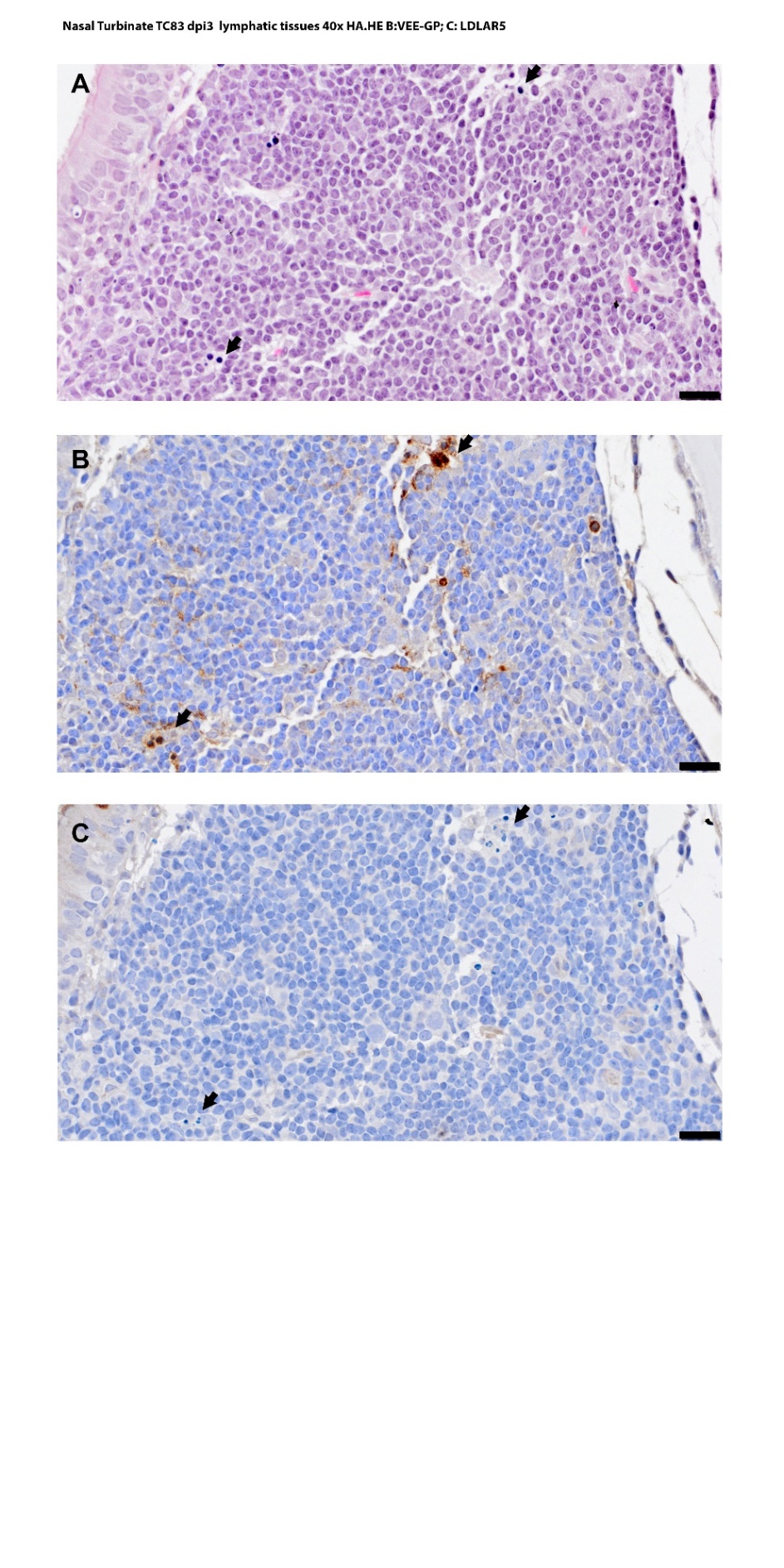
**

**Figure S4. VEEV GP and LDLRAD3 distribution in nasal lymphatic tissues** **at three days post-infection.** Representative images which show (**A**) a H&E-stained section shows lymphatic tissue with some cells dense nuclear (arrow) spread in lymphocytes and –follicular dendritic cells, (**B**) an adjacent section to ***A*** stained with VEE-GP by IHC (arrow shows VEE-GP positive staining with same location as ***A***) and (**C**) section probed for LDLRAD3 by IHC shows no significant labeling in lymphatic tissues; arrow shows cell that was stained VEE-GP

in ***B***. Bar= 20μm.

**
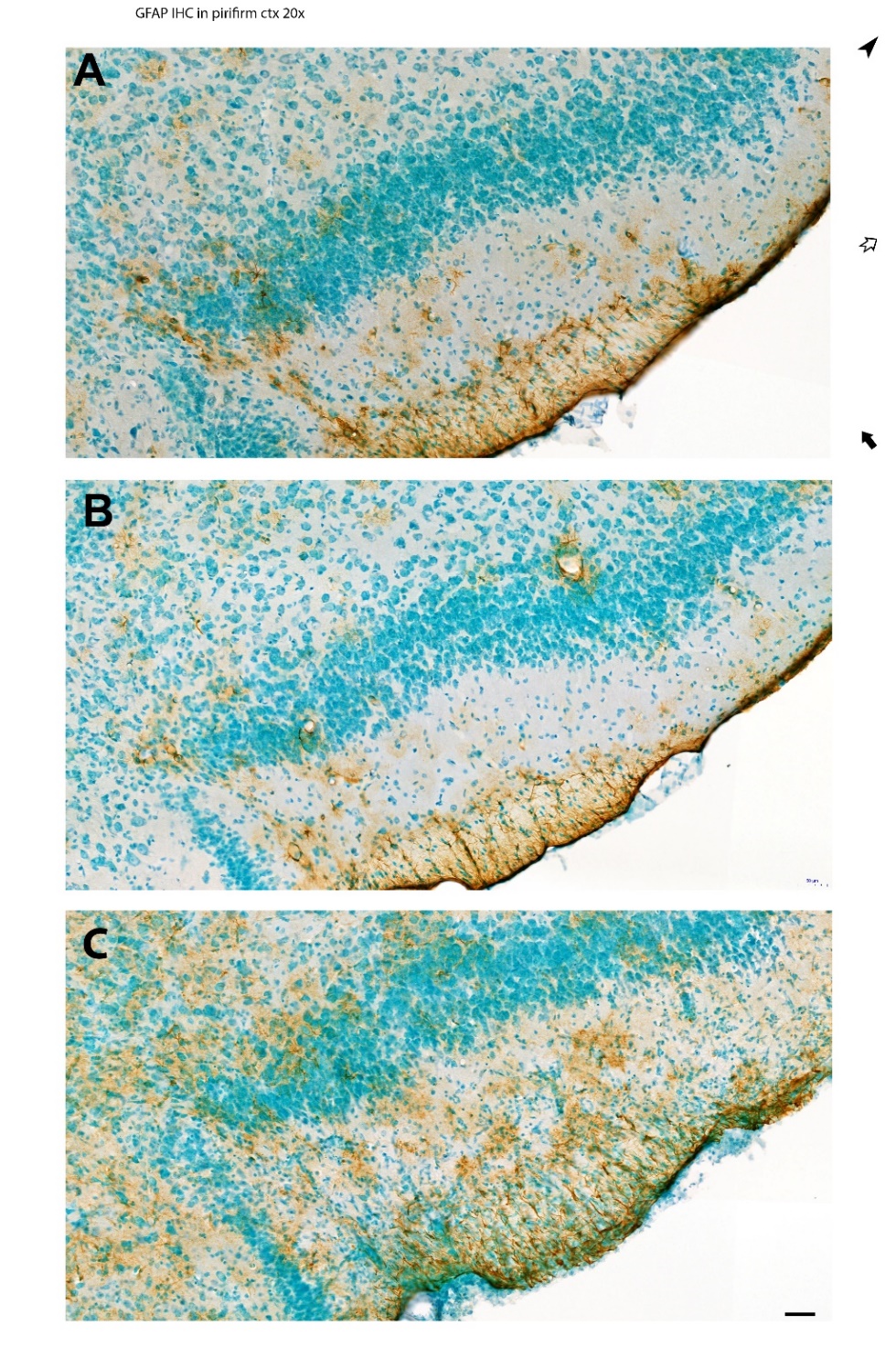
**

**Figure S5. Immunohistochemistry of GFAP in piriform cortex in infected and noninfected brain tissues.**  (**A**) An image from a PBS inoculated mouse which illustrated GFAP expression in normal piriform cortex. (**B**) An image from a VEEV TC-83 infected mouse brain on 2 dpi which has minimum changes. (**C**) An image from an infected mouse brain on dpi 6 which shows an increase in GFAP along the lateral ventricle, the gray matter of cortex, and meninges. Bar=50μm.

**
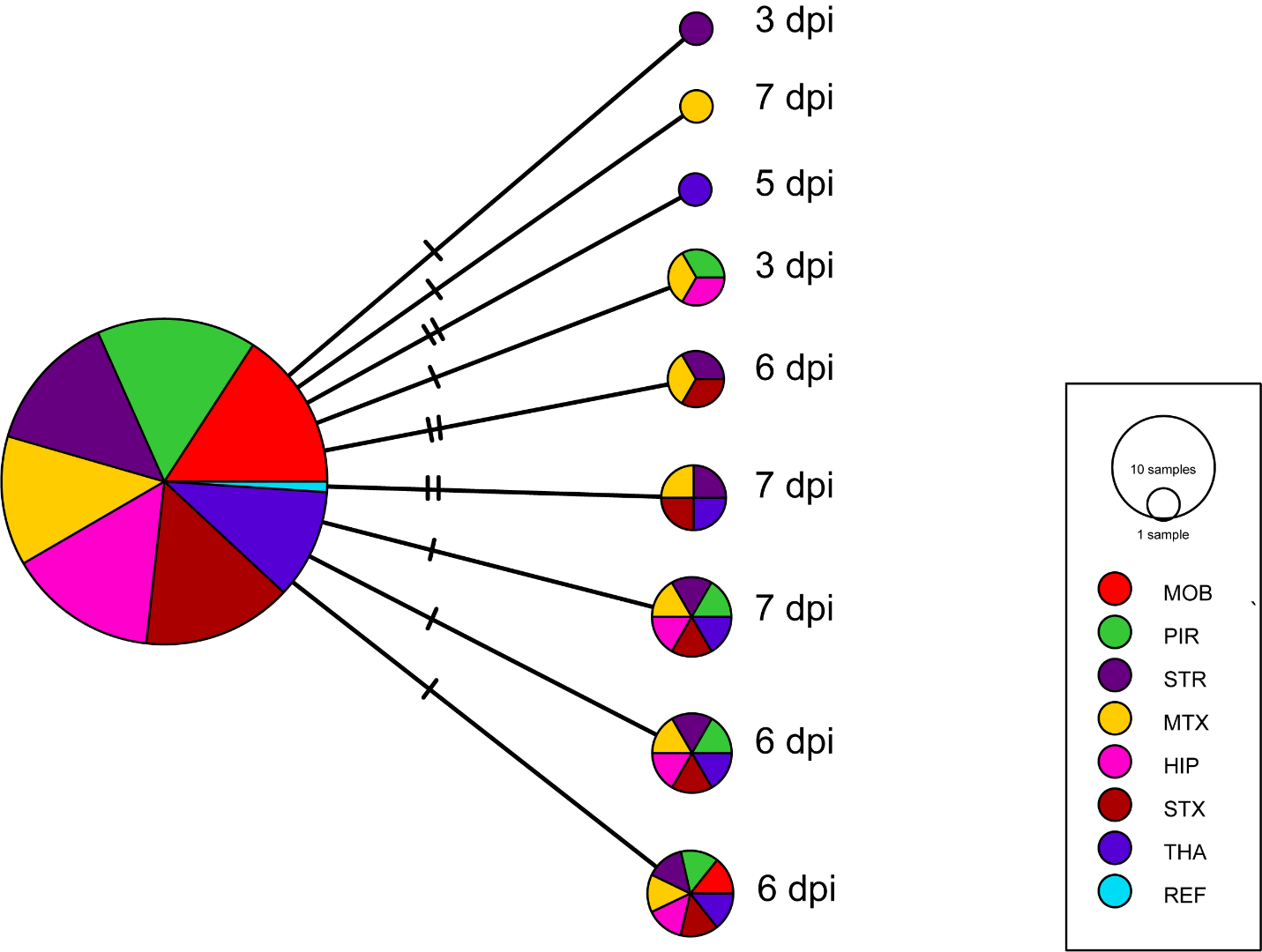
Figure Figure S6. Spatial network analysis of the genome consensus sequences of VEEV TC-83 in seven areas of the mouse brain.** A network analysis of full-length genomes of VEEV TC-83 from the RNA-Seq samples from 3-,5-, 6-, and 7-days post-infection (dpi) was conducted using PopArt. Some samples were omitted from analysis due to low genome coverage. These included all samples from the cerebellum, three samples from thalamus at 1 dpi, three from the main olfactory bulb at 7 dpi and one sample from hippocampus at 7 dpi. Hatch marks indicate the number of nucleotide differences between viral genome consensus sequences. Abbreviations: dpi-days post-infection; MOB-main olfactory bulb; PIR-piriform cortex; STR-striatum; MTX-motor cortex; HIP-hippocampus; THA-thalamus; CBX-cerebellum; REF-reference genome.

**Table S1. Nonsynonymous (Ns) mutations of VEEV TC-83 identified at 3-, 5-, 6-, and 7- days post-infection in the brains of intranasally-infected mice.**

| Nucleotide Position | Amino Acid Change | Gene | Times observed | Unique times observed | DPI | Nucleotide Change | Brain Portion | Frequency Range (%) | Frequency Average (%) | Frequency Median (%) |
| --- | --- | --- | --- | --- | --- | --- | --- | --- | --- | --- |
| 93 | Ala17Thr | NSP1 | 1 | 1 | 6 | G -> A | STX | 1.7 | 1.7 | 1.7 |
| 355 | Cys104Tyr | NSP1 | 2 | 1 | 3 | G -> A | MOB, PIR | 2.2-5.6 | 3.9 | 3.9 |
| 421 | Pro126Leu | NSP1 | 6 | 1 | 5 | C -> T | PIR, STR, MTX, HIP, STX, THA | 4.3-32.5 | 16.8 | 12.9 |
| 1362 | Ile440Val | NSP1 | 4 | 1 | 5 | A -> G | STR, HIP, MTX, STX | 3.5-14.8 | 9.2 | 9.3 |
| 1581 | Leu513Val | NSP1 | 1 | 1 | 3 | T -> G | PIR | 3.1 | 3.1 | 3.1 |
| 1631 | Met529Ile | NSP1 | 2 | 1 | 5 | G -> A | MTX, HIP | 2.0-4.3 | 3.1 | 3.1 |
| 1663 | Thr5Lys | NSP2 | 4 | 1 | 6 | C -> A | PIR, HIP, STX, THA | 14.3-23.2 | 19.3 | 19.9 |
| 1665 | Pro6Ser | NSP2 | 1 | 1 | 6 | C -> T | THA | 1.4 | 1.4 | 1.4 |
| 1969 | Ala107Val | NSP2 | 5 | 1 | 5 | C -> T | PIR, STR, MTX, STX, THA | 9.5-30.9 | 19.2 | 17.7 |
| 2161 | Thr171Ile | NSP2 | 4 | 1 | 5 | C -> T | PIR, STR, MTX, STX | 3.5-10.4 | 5.9 | 4.9 |
| 2194 | Ile182Thr | NSP2 | 3 | 1 | 6 | T -> C | MTX, STX, THA | 3.0-3.2 | 3.7 | 3.2 |
| 2281 | Lys211Arg | NSP2 | 2 | 1 | 5 | A -> G | MTX, STX | 7.9-9.1 | 8.5 | 8.5 |
| 2507 | Phe286Leu | NSP2 | 3 | 2 | 6 | T -> A | STR, MTX, STX | 1.0-1.3 | 1.1 | 1.0 |
| 2617 | Leu323Trp | NSP2 | 4 | 1 | 7 | T -> G | STR, MTX, STX, THA | 28.4-47.5 | 38.4 | 39.0 |
| 2627 | Asp326Glu | NSP2 | 12 | 5 | 3 | C -> A | MOB, PIR, STR, MTX, HIP, STX, THA | 1.1-41.2 | 12.9 | 3.3 |
| 2719 | Thr357Ile | NSP2 | 2 | 2 | 6 | C -> T | MTX, THA | 2.0-2.2 | 2.1 | 2.1 |
| 2875 | Val409Glu | NSP2 | 1 | 1 | 6 | T -> A | STR | 1.1 | 1.1 | 1.1 |
| 2947 | Thr433Ile | NSP2 | 3 | 1 | 6 | C -> T | PIR, MTX, STX | 11.7-18.3 | 15.1 | 15.2 |
| 2977 | Thr443Ile | NSP2 | 1 | 1 | 7 | C -> T | STX | 6.7 | 6.7 | 6.7 |
| 2985 | Ile446Leu | NSP2 | 1 | 1 | 5 | A -> C | PIR | 12.0 | 12.0 | 12.0 |
| 3039 | Pro464Ser | NSP2 | 6 | 1 | 6 | C -> T | PIR, STR, MTX, HIP, STX, THA | 9.1-32.7 | 23.2 | 23.9 |
| 3045 | Pro466Thr | NSP2 | 2 | 1 | 5 | C -> A | MTX, STX | 8.3-10.6 | 9.4 | 9.4 |
| 3052 | Asp468Gly | NSP2 | 1 | 1 | 3 | A -> G | STR | 5.3 | 5.3 | 5.3 |
| 3070 | Ala474Val | NSP2 | 4 | 1 | 6 | C -> T | STR, MTX, STX, THA | 10.4-26.9 | 16.9 | 15.2 |
| 3139 | Gln497Arg | NSP2 | 1 | 1 | 3 | A -> G | MOB | 2.6 | 2.6 | 2.6 |
| 3154 | Asp502Val | NSP2 | 7 | 3 | 3; 5 | A -> T | MOB, PIR, STR, MTX, HIP, STX, THA | 1.5-48.3 | 17.1 | 12.7 |
| 3156 | Tyr503His | NSP2 | 1 | 1 | 7 | T -> C | STX | 5.7 | 5.7 | 5.7 |
| 3340 | Arg564His | NSP2 | 1 | 1 | 3 | G -> A | MOB | 1.3 | 1.3 | 1.3 |
| 3372 | Arg575Trp | NSP2 | 1 | 1 | 3 | C -> T | PIR | 2.6 | 2.6 | 2.6 |
| 3396 | Tyr583His | NSP2 | 4 | 1 | 5 | T -> C | PIR, STR, HIP, STX | 7.8-14.3 | 10.0 | 8.8 |
| 3409 | Thr587Asn | NSP2 | 3 | 1 | 7 | C -> A | STR, MTX, STX | 15.4-63.2 | 32.9 | 20.1 |
| 3414 | Thr589Ser | NSP2 | 2 | 1 | 3 | A -> T | MOB, PIR | 6.3-7.1 | 6.7 | 6.7 |
| 3433 | Pro595Gln | NSP2 | 3 | 1 | 7 | C -> A | STR, MTX, STX | 86.2-90.9 | 89.2 | 90.4 |
| 3463 | Arg605Ile | NSP2 | 6 | 1 | 6 | G -> T | PIR, STR, MTX, HIP, STX, THA | 52.5-78.7 | 63.5 | 60.7 |
| 3556 | Val636Ala | NSP2 | 2 | 1 | 5 | T -> C | PIR, STR | 3.3-4.6 | 4.0 | 4.0 |
| 3678 | Ile677Leu | NSP2 | 3 | 1 | 7 | A -> T | STR, MTX, STX | 11.2-37.5 | 20.3 | 12.1 |
| 3958 | Thr770Met | NSP2 | 7 | 2 | 5 | C -> T | MOB, PIR, STR, MTX, HIP, STX, THA | 4.9-60.5 | 24.9 | 21.6 |
| 4081 | Gly17Glu | NSP3 | 1 | 1 | 5 | G -> A | PIR | 1.8 | 1.8 | 1.8 |
| 4386 | Asp119Asn | NSP3 | 5 | 1 | 5 | G -> A | PIR, STR, MTX, HIP, STX, THA | 2.2-11.4 | 5.8 | 4.9 |
| 4513 | Ala161Val | NSP3 | 2 | 1 | 3 | C -> T | MOB, PIR | 2.2-9 | 5.6 | 5.6 |
| 4878 | Ala283Thr | NSP3 | 3 | 1 | 6 | G -> A | PIR, MTX, STX | 10.0-18.0 | 14.2 | 14.6 |
| 4990 | Val320Ala | NSP3 | 8 | 1 | 6 | T -> C | MOB, PIR, STR, MTX, HIP, STX, THA, CBX | 56.8-93.1 | 80.9 | 87.1 |
| 4999 | Tyr323Cys | NSP3 | 6 | 1 | 6 | A -> G | MOB, STR, MTX, HIP, STX, THA | 5.5-81.6 | 43.5 | 43.2 |
| 5025 | Glu332Lys | NSP3 | 1 | 1 | 7 | G -> A | MTX | 2.5 | 2.5 | 2.5 |
| 5029 | Thr333Ile | NSP3 | 6 | 2 | 3 | C -> T | MOB, PIR, STR, HIP, STX | 2.9-64.2 | 22.6 | 18.3 |
| 5035 | Pro335Leu | NSP3 | 1 | 1 | 5 | C -> T | HIP | 1.3 | 1.3 | 1.3 |
| 5052 | Glu341Gln | NSP3 | 1 | 1 | 5 | G -> C | HIP | 1.9 | 1.9 | 1.9 |
| 5082 | Gly351Arg | NSP3 | 1 | 1 | 7 | G -> A | STX | 6.2 | 6.2 | 6.2 |
| 5185 | Leu385Gln | NSP3 | 5 | 1 | 5 | T -> A | MOB, STR, MTX, HIP, STX | 1.5-3.7 | 2.5 | 2.0 |
| 5257 | Ser409Leu | NSP3 | 5 | 1 | 6 | C -> T | PIR, MTX, HIP, STX, THA | 6.6-18.5 | 11.9 | 10.9 |
| 5277 | Ala416Thr | NSP3 | 4 | 1 | 5 | G -> A | PIR, STR, HIP, STX | 6.1-14.5 | 8.4 | 6.5 |
| 5365 | Asn445Ser | NSP3 | 5 | 1 | 5 | A -> G | MOB, PIR, STR, MTX, HIP, STX | 1.6-4.1 | 2.6 | 1.8 |
| 5421 | Thr464Ala | NSP3 | 2 | 1 | 6 | A -> G | HIP, STX | 2.0-2.2 | 2.1 | 2.1 |
| 5470 | Ser480Leu | NSP3 | 1 | 1 | 5 | C -> T | HIP | 2.2 | 2.2 | 2.2 |
| 5482 | Ser484Ile | NSP3 | 1 | 1 | 7 | G -> T | MTX | 1.2 | 1.2 | 1.2 |
| 5553 | Leu508Phe | NSP3 | 4 | 1 | 7 | C -> T | STR, MTX, STX, THA | 62.1-84.6 | 73.3 | 73.2 |
| 6188 | Ser162Arg | NSP4 | 1 | 1 | 3 | T -> G | MOB | 1.1 | 1.1 | 1.1 |
| 6261 | Pro187Ser | NSP4 | 1 | 1 | 7 | C -> T | MTX | 5.9 | 5.9 | 5.9 |
| 6475 | Lys258Thr | NSP4 | 10 | 7 | 6; 7 | A -> C | PIR, STR, HIP, MTX, THA, STX | 2.4-6.3 | 3.8 | 3.6 |
| 6558 | Val286Leu | NSP4 | 1 | 1 | 5 | G -> T | THA | 2.7 | 2.7 | 2.7 |
| 6576 | Asp292Asn | NSP4 | 1 | 1 | 3 | G -> A | PIR | 2.3 | 2.3 | 2.3 |
| 6939 | Ile413Leu | NSP4 | 7 | 1 | 5 | A -> T | MOB, PIR, STR, MTX, HIP, STX, THA | 2.2-25.6 | 12.2 | 13.8 |
| 6942 | His414Tyr | NSP4 | 1 | 1 | 5 | C -> T | MOB | 1.9 | 1.9 | 1.9 |
| 7252 | Thr517Ile | NSP4 | 1 | 1 | 5 | C -> T | MOB | 4.6 | 4.6 | 4.6 |
| 7386 | Leu562Phe | NSP4 | 1 | 1 | 5 | C -> T | THA | 2.3 | 2.3 | 2.3 |
| 7395 | Leu565Val | NSP4 | 1 | 1 | 7 | C -> G | STR | 1.9 | 1.9 | 1.9 |
| 7593 | Gln11Leu | Capsid | 2 | 1 | 7 | A -> T | STR, STX | 2.3-4.9 | 3.6 | 3.6 |
| 7614 | Pro18Leu | Capsid | 1 | 1 | 3 | C -> T | STR | 2.7 | 2.7 | 2.7 |
| 7620 | Ala20Val | Capsid | 1 | 1 | 5 | C -> T | HIP | 1.3 | 1.3 | 1.3 |
| 7677 | Gln39Leu | Capsid | 1 | 1 | 3 | A -> T | PIR | 2.5 | 2.5 | 2.5 |
| 7694 | Met45Leu | Capsid | 2 | 1 | 7 | A -> T | PIR, STX | 2.4-15.1 | 8.7 | 8.7 |
| 7733 | Pro58Ser | Capsid | 1 | 1 | 5 | C -> T | MOB | 2.4 | 2.4 | 2.4 |
| 7779 | Lys73Ile | Capsid | 1 | 1 | 7 | A -> T | MTX | 3.5 | 3.5 | 3.5 |
| 7787 | Gly76Arg | Capsid | 14 | 3 | 3; 6 | G -> A | MOB, PIR, STR, MTX, HIP, STX, THA | 1.6-30.9 | 13.2 | 9.8 |
| 7923 | Lys121Arg | Capsid | 1 | 1 | 3 | A -> G | MOB | 2.3 | 2.3 | 2.3 |
| 7949 | Ile130Phe | Capsid | 12 | 8 | 6; 7 | A -> T | MOB, PIR, STR, HIP, STX, THA | 1.1-1.3 | 1.2 | 1.2 |
| 7954 | Met131Ile | Capsid | 1 | 1 | 7 | G -> T | STX | 1.1 | 1.1 | 1.1 |
| 8168 | His203Tyr | Capsid | 1 | 1 | 3 | C -> T | MOB | 2.0 | 2.0 | 2.0 |
| 8615 | Arg18Gly | E2 | 4 | 1 | 3 | A -> G | MOB, PIR, STR, HIP | 1.3-5.3 | 2.4 | 1.6 |
| 8801 | His80Tyr | E2 | 1 | 1 | 6 | C -> T | MOB | 1.1 | 1.1 | 1.1 |
| 8924 | His121Tyr | E2 | 6 | 1 | 5 | C -> T | MOB, PIR, STR, MTX, HIP, STX | 1.7-39.1 | 10.6 | 4.4 |
| 9144 | Thr194Ile | E2 | 1 | 1 | 5 | C -> T | MOB | 1.3 | 1.3 | 1.3 |
| 9507 | Ala315Val | E2 | 1 | 1 | 6 | C -> T | STR | 1.1 | 1.1 | 1.1 |
| 9618 | Pro352Leu | E2 | 2 | 1 | 6 | C -> T | STR, MTX, HIP | 1.1-5.6 | 2.9 | 1.9 |
| 9672 | Gly370Val | E2 | 1 | 1 | 6 | G -> T | PIR | 1.5 | 1.5 | 1.5 |
| 9701 | Val380Phe | E2 | 1 | 1 | 7 | G -> T | STX | 1.0 | 1.0 | 1.0 |
| 9717 | Ser385Phe | E2 | 5 | 1 | 7 | C -> T | PIR, STR, MTX, HIP, STX | 12.0-43.4 | 21.6 | 19.4 |
| 9765 | Arg401Gln | E2 | 1 | 1 | 5 | G -> A | PIR | 2.0 | 2.0 | 2.0 |
| 9791 | Phe410Leu | E2 | 1 | 1 | 5 | T -> C | MOB | 1.4 | 1.4 | 1.4 |
| 10137 | Tyr46Cys | E1 | 31 | 11 | 3; 5; 6; 7 | A -> G | MOB, PIR, STR, MTX, HIP, STX, THA | 1.0-4.0 | 1.4 | 1.2 |
| 10139 | Val47Ile | E1 | 7 | 1 | 5 | G -> A | MOB, PIR, STR, HIP, STX, THA | 1.6-10 | 4.4 | 3.0 |
| 10422 | Ser141Phe | E1 | 1 | 1 | 6 | C -> T | STX | 2.4 | 2.4 | 2.4 |
| 10481 | Ile161Leu | E1 | 6 | 1 | 7 | A -> T | PIR, HIP, MTX, STX, THA | 22.0-40.0 | 31.9 | 32.5 |
| 10826 | Ile276Leu | E1 | 3 | 1 | 5 | A -> C | MOB, HIP, STX | 1.5-2.1 | 1.7 | 1.7 |
| 10844 | Ile282Val | E1 | 1 | 1 | 5 | A -> G | THA | 1.3 | 1.3 | 1.3 |
| 10874 | Glu292Gln | E1 | 1 | 1 | 3 | G -> C | STR | 3.5 | 3.5 | 3.5 |
| 10997 | Pro333Ser | E1 | 2 | 1 | 3 | C -> T | PIR, STR | 3.1-4.6 | 3.9 | 3.9 |
| 11028 | Ala343Val | E1 | 1 | 1 | 6 | C -> T | MOB | 1.9 | 1.9 | 1.9 |
| 11143 | His381Gln | E1 | 1 | 1 | 3 | C -> A | STR | 4.8 | 4.8 | 4.8 |
| 11183 | Ala395Ser | E1 | 4 | 1 | 7 | G -> T | PIR, HIP, STX, THA | 2.6-10.6 | 6.0 | 5.4 |
| 11232 | Thr411Ile | E1 | 4 | 1 | 7 | C -> T | STR, MTX, STX, THA | 1.3-7.5 | 3.9 | 3.3 |
| 11294 | Ala432Thr | E1 | 1 | 1 | 6 | G -> A | STX | 1.5 | 1.5 | 1.5 |
